# The Role of Non-animal Origin Feed Ingredients in Transmission of Viral Pathogens of Swine: A Review of Scientific Literature

**DOI:** 10.3389/fvets.2019.00273

**Published:** 2019-08-22

**Authors:** Rebecca K. Gordon, Ingrid K. Kotowski, Kari F. Coulson, Donald Link, Alexandra MacKenzie, Joyce Bowling-Heyward

**Affiliations:** ^1^U.S. Department of Agriculture, Animal and Plant Health Inspection Service, Veterinary Services, Raleigh, NC, United States; ^2^U.S. Department of Agriculture, Animal and Plant Health Inspection Service, Veterinary Services, Riverdale, MD, United States

**Keywords:** feed, ingredient, virus, pathogen, transmission, contaminated, swine

## Abstract

The emergence of porcine epidemic diarrhea (PED) in commercial swine in North America and growing concerns about the potential for the introduction of African swine fever (ASF) from China, the European Union, or other affected regions has put a spotlight on the possible role of contaminated feed and feed ingredients in the introduction and transmission of viral swine pathogens. This paper systematically reviews the scientific literature regarding whether non-animal origin ingredients of commercial swine feed could introduce or transmit viral pathogens of swine into or within the United States. The purpose of this review is to identify, evaluate, and summarize the relevant scientific knowledge, published through March 2018, and to identify information gaps and research needs, thereby making the available evidence more accessible to policy makers, the swine industry, and the scientific community. A total of 26 documents were selected for the final review process, which included experimental studies, case reports, epidemiological investigations, and scientific opinion, among others. The review found that the scientific literature has addressed some critical experimental questions pertaining to transmission of swine viruses via feed and feed ingredients, but the current body of scientific knowledge lacks conclusive evidence of virus contamination of non-animal origin feed ingredients of commercial swine feed, particularly for imported commodities, and further investigation into the epidemiology of virus transmission via feed to swine under field conditions through natural feeding behavior is warranted. Additional studies of how imported ingredients of commercial swine feed are sourced, processed, transported and, thus, contaminated prior to importation into the United States are needed. Moving forward, studies designed to examine the likely source(s) of contamination and subsequent virus mitigation steps in processing and post-processing may be the most fruitful focus of research.

## Introduction

### Rationale

Over the past three decades, the swine industry in the United States has experienced several significant disease outbreak events with highly pathogenic viral pathogens, including porcine reproductive and respiratory syndrome virus (PRRSV), porcine circovirus type 2 (PCV2), and, most recently, the swine enteric coronaviruses, including porcine delta coronavirus (PDCoV) and porcine epidemic diarrhea virus (PEDV) (1–4). These disease events have resulted in significant clinical consequences with increased morbidity and mortality, in some cases reaching 100%, as well as economic devastation to the swine industry with financial losses estimated in the hundreds of millions to billions of dollars (1, 5). Among other shared characteristics, all three causative agents had been previously known to cause mild or non-pathogenic disease in swine prior to the re-emergence event (4). Additionally, emerging and/or novel diseases that present with common clinical signs consistent with expected production diseases may be present yet remain undetected for some time, contributing to widespread transmission amongst the industry and hindering local containment. Thus, once identified, the unforeseen emergence of a disease outbreak with high morbidity, high mortality, and rapid, transboundary spread brings about fundamental questions regarding the epidemiology and origin of the agent as well as an immediate need for both short- and long-term response activities and mitigation strategies to control the outbreak and prevent future events (4).

During the 2013–2014 outbreak of porcine epidemic diarrhea (PED) in North America, contaminated feed and feed ingredients were suspected as a potential introduction and/or transmission route for spread as early cases of PED in Canada were associated with a common feed source containing spray-dried porcine plasma (SDPP) (6–9). Additionally, genetic and phylogenetic analyses revealed that United States (U.S.) strains of PEDV were closely related to Chinese strains, particularly the 2012 strain from Anhui Province in China (10), fueling concerns that imported, contaminated commodities from China may have been the route of introduction into the United States. Growing anecdotal evidence and early investigative studies (11) have further implicated feed and feed ingredients as the possible transmission vehicle for PEDV although the source(s) and route(s) of introduction have not been definitively identified. Compounded by the recent outbreaks of African swine fever (ASF) in Asia and Europe^1^, there is rising concern that contaminated imported commodities, particularly non-animal origin ingredients of commercial swine feed, could introduce and transmit viral pathogens of significant concern to the U.S. swine industry.

Despite extensive investigative work in the field and the laboratory, the specific mode of introduction of exotic viral pathogens such as PEDV into the United States and, subsequently, into domestic swine premises remains unknown. In order for feed or feed ingredients to be a route of disease introduction into the United States, they must become contaminated with the causative agent; avoid inactivation through (trans-oceanic) transport, feed manufacturing, processing, and distribution; and be ingested at a dose sufficient to cause infection in a susceptible pig.

The likelihood of disease transmission via contaminated feed and feed ingredients is non-zero as pathogens such as *Salmonella* are known to be transmitted via swine feed (12, 13). However, accurate estimates of the risk that contaminated non-animal origin feed and feed ingredients pose in the introduction and subsequent transmission of PEDV and other exotic swine viruses are not available, particularly in comparison to other recognized risk factors such as movement of infected pigs, transport vehicles, personnel, and waste feeding of unprocessed or improperly processed animal products. Thus, making sound decisions regarding risk mitigation measures in the face of an uncertain risk is challenging.

The emergence of PEDV in North America and growing concerns about the potential for introduction of ASF from China, the European Union, or other affected regions has put a spotlight on the possible role of contaminated feed and feed ingredients in the introduction and transmission of viral swine pathogens. The characteristics of modern swine production—globalization of trade, including significant increases in the volume of imported bulk feed ingredients, intensification and vertical integration of production, and extensive movement of pigs and related production components (e.g., transport vehicles, feed, personnel)—and the occurrence of emerging swine diseases in new geographic ranges (e.g., ASF) and/or with increased pathogenicity (e.g., PED) suggests that the critical production inputs along with existing biosecurity and mitigating measures that have historically delivered an acceptable level of protection may need to be re-evaluated.

### Objective

In order to better inform policy makers, swine industry stakeholders, and the scientific community, a literature review was conducted on the scientific evidence regarding whether non-animal origin ingredients of commercial swine feed could introduce or transmit viral pathogens of swine into or within the United States. The goal of the literature review is to understand the current scientific knowledge and to identify information gaps. The results may support future scientific research for evaluating the risk of entry of exotic viral pathogens via specific feed ingredients from source countries and mitigation measures to prevent exposure to U.S. swine populations.

### Research Question

This literature review aims to answer the following research question:

What evidence is available in published scientific literature regarding whether non-animal origin ingredients of commercial swine feed could transmit viral pathogens of swine into or within the United States?

## Methods of the Literature Review

The methodology of this literature review follows the framework of a qualitative systematic review (14). The literature review aims to identify, evaluate, and summarize the findings of relevant research studies, thereby making the available evidence more accessible to decision makers, other stakeholders, and the scientific community. When appropriate, combining the results of several studies can give a more reliable and precise estimate of the available knowledge, intervention, or control measures' effectiveness than one study alone. The methodology of the literature review has four main components: (1) identifying and selecting research evidence, (2) data extraction and quality assessment, (3) data synthesis, and (4) report writing (14).

### Literature Search and Study Selection

A search of the National Library of Medicine/PubMed, National Agricultural Library/PubAg, National Agricultural Library/Navigator (including major databases: AGRICOLA, AGRIS, BIOSIS, CABI, EBSCO Environment Complete, GEOBASE, GeoRef, MEDLINE, Scopus, Web of Science, and Zoological Record) and Google Scholar was conducted to identify published scientific literature pertaining to evidence regarding whether non-animal origin ingredients of commercial swine feed could transmit viral pathogens of swine into or within the United States. Studies published any time through March 2018 were identified.

**Figure 1:**
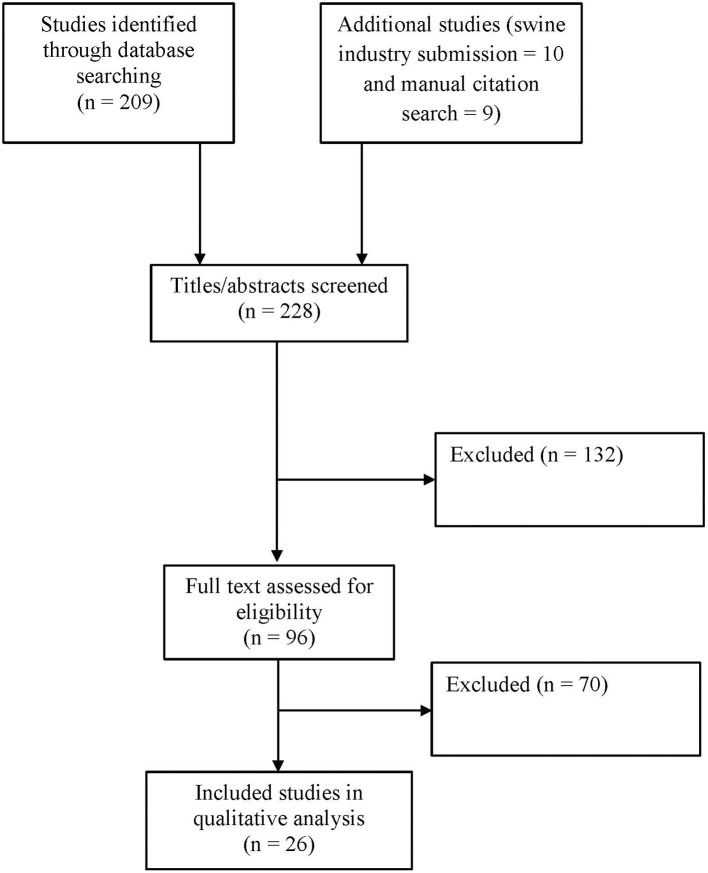
Overview flow chart of study selection process.

The study selection process was performed in two stages. In the first stage, an initial screening of search results was performed based on title and abstract. In the second stage, the full text of the preliminary list of studies was evaluated. Additional articles were obtained through manual review of reference citations in the relevant literature. Figure 1 provides an overview of the study selection process. Studies were excluded that did not meet the purpose statement of the literature review. Studies excluded after a full text review are reported in Data Sheet S1. Reasons for exclusion included:

- Focus on non-viral pathogens
- Focus on disease transmission routes other than feed and feed ingredients
- Animal origin feed ingredients
- Focus on mitigations/treatment/disinfection of equipment or sanitizing feed
- No English translation available
- Full text not available
- Duplicate publication
- Subject matter outside the scope of the review.

The most common reasons for exclusion, in descending order, were: focus on disease transmission routes other than feed or feed ingredients; focus on mitigations/treatment/disinfection of equipment or sanitizing feed; subject matter outside the scope of the review; and focus on animal origin feed ingredients. The two-stage study selection resulted in retention of 26 included studies. The studies included for the qualitative analysis are reported in Data Sheet S2.

### Data Extraction and Quality Assessment

The data extraction component is the process by which research reviewers obtain the necessary information about study characteristics, methods, and findings from the included studies. The quality assessment component aims to identify internal and external validity of the selected studies. Standardized data extraction provides consistency in a literature review, thereby reducing potential bias and improving validity and reliability (14).

A fillable, pdf data extraction form template was created for the purpose of this literature review. Twenty-six published articles were included in the data extraction and quality assessment process. Reviewers worked in pairs to perform the data extraction and quality assessment. Each article was reviewed for general information, study characteristics, and outcome results. For the quality assessment portion, the reviewers reported on potential sources of bias, shortfalls in the statistical and analyses methodology, the quality of reporting, and the generalizability of the study to the commercial swine industry in the United States. The data extraction form template is provided in the supplementary information Figure S1.

### Data Synthesis and Report Writing

A qualitative, narrative approach was used for the data synthesis and report writing. The information extracted in the data extraction process was summarized into a data synthesis table. This table is provided in Table S1. From the data synthesis table, relevant information from the individual studies was collated and summarized into a report.

## Literature Review Results

The 26 studies included in this literature review were collated into three categories: background information on risk factors for transmission and virus survival on fomites, epidemiology and outbreak investigations, and experimental studies with swine bioassays. Information extracted from these studies may overlap into more than one category and, thus, may be discussed in more than one section. Additionally, the study summaries provided in this section are not meant to be detailed and fully comprehensive but rather are focused on the information pertinent to this literature review, namely, non-animal origin feed ingredients.

### Risk Factors for Transmission and Virus Survival on Fomites

Numerous investigators have reviewed the epidemiology and impact of swine viral pathogens and analyzed industry expert opinion and outbreak information to identify risk factors for transmission of swine pathogens. Additionally, several studies have looked at the survivability of viruses on various fomites related to swine production and husbandry, including feed and feed ingredients. Methods used by these investigators include expert elicitation, questionnaire-based *post-hoc* outbreak investigations, and experimental studies of the survival of viruses in contact with fomites. The studies are grouped into two categories: risk factors for virus transmission and virus survival in contact with fomites. The virus scope of these studies includes African swine fever virus (ASFV), porcine high fever disease virus, highly pathogenic PRRSV, pseudorabies virus (PRV), Aujeszky's disease virus, and blue eye disease virus.

#### Risk Factors for Virus Transmission

Three studies examined the epidemiology of specific swine pathogen(s) or outbreak events in various regions of the world (15–17). The pathogens covered include porcine high fever disease virus in Vietnam and ASFV in Nigeria and Eastern Europe. One study used expert elicitation methods to identify risk factors for transmission of highly pathogenic porcine reproductive and respiratory syndrome (PRRS) into and within Australia (18).

Le et al. (16) conducted a retrospective survey to identify risk factors that may have contributed to the spread of porcine high fever disease (PHFD) from China to Vietnam in 2008. PHFD is considered a PRRS-related syndrome. Using a survey administered to individual households in a southern province of Vietnam, investigators found that, among other things, the use water green crop as pig feed and the presence of ducks, with or without direct contact with pigs were positively associated with clinical signs consistent with PHFD in swine. Water green crop are fed directly to pigs after harvest, without processing. The authors hypothesize that ducks potentially amplify the virus, similar to data shown for PRRSV, and contaminate the water green crop fed to pigs. It is also believed that porcine high fever disease virus can persist in water, further contaminating the greens before harvest. The findings of this study suggest that unprocessed non-animal origin feed ingredients could be contaminated with virus by other animals with subsequent transmission to swine (16).

A retrospective case-control study by Fasina et al. (15) sought to identify risk factors associated with ASF outbreaks in Nigerian swine herds. An epidemiological questionnaire was administered to all participating farm owners and a number of factors associated with increased risk of ASF were identified, including: purchase of untested pigs from neighboring farms, presence of an abattoir in the community, wild bird access to pig pens, sharing of equipment between farms, and unprotected feed sources (rodent access). The final logistical regression model showed that protecting feed and water sources, separation of sick and healthy pigs, and washing/disinfecting equipment were negatively associated (protective) with ASF infection. These findings indicate that preventing rodent access to feed sources and the use of commercial feeds, as opposed to swill feeding, were potentially protective measures in the farms studied (15).

A review by Guinat et al. (17) summarizes findings of ASFV transmission studies performed in Eastern European and Baltic countries. In respect to feed-to-pig transmission pathways, the authors referenced a European Commission (2014) epidemiological report in Latvia and Lithuania suggesting that fresh grass and seeds may have been contaminated by wild boar feces containing ASFV and transmitted the virus to domestic backyard pigs. A similar study focused on ASF in Latvia also suggested that feeding potentially contaminated (via wild boar) fresh grass or crops was a risk factor for ASF occurrence in backyard holdings; however, swill feeding could not be excluded as a source (19). A study conducted in Kenya in 1921 demonstrated that ASFV could be transmitted to pigs when they consumed infected feces and urine but failed to transmit when contaminated sweet potatoes or bananas were consumed (20). While several references were provided for documented transmission of ASFV to domestic pigs through feeding of swine-origin feed ingredients, there is limited data on the relationship of ASFV transmission and non-animal origin feed ingredients (17).

Brookes et al. (18) elicited industry expert opinions to identify entry and exposure routes with the highest probability of occurrence for introduction of highly pathogenic PRRS from south-east Asia to Australia. Pig industry experts attending the Australian Pig Veterinarians' Annual Conference in Melbourne, Australia in June 2013 were given a questionnaire and asked to indicate the probability of occurrence of 28 entry routes and 36 exposure routes within fixed probability ranges over 1 year. There was significant agreement on 16 entry routes; the entry routes with the highest probability of occurrence and statistically significant agreement among participants included entry of highly pathogenic PRRS by humans or animal feed acting as fomites (traveling or being shipped by air) and raw pork entering through the postal service or by a private individual (18). There was statistically significant agreement on 29 exposure routes; the routes with the highest estimated probability of occurrence all involved disposal of waste to feral or backyard pigs. The highest probability exposure route for commercial pigs was thought to be contact with a human acting as a fomite or access to animal feed/additives from south-east Asia.

#### Virus Survival in Contact With Fomites

Three studies examined virus survivability on various swine-related fomites, including feed and feed ingredients (21–23). The viruses covered by these studies were PRV, PRRSV, Aujeszky's disease virus, and blue eye disease virus.

Schoenbaum et al. (21) conducted an experimental study to investigate the survival duration of PRV in contact with various solid and liquid fomites commonly found in swine production environments. Feed or feed ingredients included in the study were green grass, whole corn, pelleted feed, and alfalfa. The authors inoculated various solid and liquid fomites with mixed stock PRV and incubated the samples at room temperature for up to 14 days. Virus activity was assessed through a cell culture based assay. They found that, in general, the quantity of infectious virus decreased over time. Of the feed or feed ingredients included in the study, the combination of PRV/saline/whole corn remained infectious the longest at 7 days with an estimated half-life of 36.3 h. The durations of infectiousness of the other combinations were shorter, ranging from 1 to 4 days with an estimated half-life of 1.0–5.1 h. The authors acknowledged that virus survival time is impacted by temperature, and speculated that virus survival times would be longer at lower temperatures and shorter at higher temperatures (21).

Pirtle and Beran (22) conducted an experimental study to investigate the survival time of PRRSV in contact with various liquid and solid fomites. Feed and feed ingredients included in the study were ground corn, pelleted swine starter feed mix, and alfalfa. The authors inoculated fomites with mixed stock PRRSV and incubated the samples at room temperature for up to 11 days. Virus activity was assessed through a cell culture based assay. In the PRRSV-spiked alfalfa sample, the authors detected PRRSV only on day 0. They did not detect any virus in the PRRSV-spiked starter feed mix and ground corn samples. The authors speculated that the pH (<7) of the samples tested and/or unknown substances present in the samples could have contributed to virus inactivation (22).

Martínez-Gamba et al. (23) conducted an experimental study to examine the persistence of bacterial and viral pathogens in feces fermented for use in animal feed (silage). Feces from 30 pigs was collected, inoculated with Aujeszky's disease virus and blue eye disease virus, and mixed with molasses and sorghum for fermentation. Flasks of ensilage were incubated at room temperature for 0, 7, 14, 28, and 56 days. Presence of virus was detected by observation of cytopathic effects on cellular monolayers and indirect immunofluorescence. Samples were positive for virus on day 0 but not at any subsequent time point. The findings indicate that fermentation is sufficient for the inactivation of the viruses tested under the experimental conditions used and could be an acceptable means for eliminating these viruses from feces used for animal feed. These results may not be applicable to viruses of other families (23).

#### Summary of Studies Regarding Risk Factors for Transmission and Virus Survival on Fomites

In summary, most of the reports and studies summarized above were not designed to focus on non-animal origin feed and feed ingredients as a potential vehicle for virus transmission; however, feed and feed ingredients were included in the study design or discussion. Similarly, some findings in this group of studies may not be generalizable to other viruses or virus families or the commercial swine industry in the United States. Given these limitations, several key points are highlighted below.

Le et al. (16) identified owning ducks and using water green crop as pig feed (together) as being associated with an increased risk of PHFD, speculating that unprocessed non-animal origin feed ingredients could be contaminated with virus by other animals with subsequent transmission to swine (16). Fasina et al. (15) identified feed and water control to be significantly associated with decreased risk of ASF infection in Nigeria, noting that feed and water biosecurity practices can prevent virus contamination by rodents and wild birds (15). Consistent with these findings, an expert opinion elicitation study conducted in Australia found industry representatives believed commercial pigs were most likely to be exposed to highly pathogenic PRRS through access to contaminated animal feed and feed additives imported from southeast Asia (18). These studies suggest that protecting unprocessed non-animal origin feed ingredients from contact with animals may decrease the likelihood of pathogen contamination. This may be particularly important for ingredients that are not processed before being fed to animals.

Several investigators have examined the persistence of viral activity in the presence of various fomites, including non-animal origin feed ingredients. In experiments in which they spiked fomites with stock virus, they found, in general, that viral activity did not persist for long periods of time under the experimental conditions used. Schoenbaum et al. (21) found that the combination of PRV/saline/whole corn remained infectious the longest at 7 days (21) while Pirtle and Beran (22) detected PRRSV activity only on day 0 in the virus-spiked alfalfa sample (22). Interestingly, Martínez-Gamba et al. (23) found that fermentation is sufficient for the inactivation of Aujeszky's disease virus and blue eye disease virus (23). Collectively, these studies suggest that, under certain experimental conditions, swine viruses can survive in non-animal origin feed ingredients.

Among the knowledge gaps identified in these studies are the need for a comprehensive evaluation of potential transmission pathways involving non-animal origin feed ingredients and swine viruses. The scientific literature regarding the survival times (including half-life estimates) for various non-animal origin feed ingredient/pathogen combinations as well as determination of infectivity is incomplete and warrants further studies along with documented replication of studies. Furthermore, the likelihood of non-animal origin feed ingredients incurring contamination and documented scenarios in which cross-contamination occurs under field conditions is unknown.

### Epidemiology and Outbreak Investigations—PEDV and PDCoV

In 2013, swine enteric coronaviruses, such as PEDV and PDCoV, emerged as pathogens of significance for the swine industry in the United States and several other countries. Efforts were made to describe the epidemiology of the outbreaks which included a focus on the sources of virus introduction and transmission. Among other things, feed and non-animal origin feed ingredients were suspected as one possible route of virus introduction and spread. Studies reviewed in this section were separated into two major categories: epidemiological reports, reviews, and surveys; and feed and feed ingredient experimental studies. These studies are primarily focused on PEDV and PDCoV; however, other swine pathogens are included such as transmissible gastroenteritis virus (TGEV), PRRSV, and PCV2.

#### Epidemiological Reports, Reviews, and Surveys

Eight epidemiological reports examining the global outbreaks of PED and PDCoV infection were reviewed. The geographic scope of these reports include Europe, Asia, and North America (4, 24–30). The authors sought to describe the outbreaks and describe associations between risk factors for transmission and disease status.

In 2014 and 2016, the European Food Safety Authority published two reports pertaining to PED and PDCoV (29, 30). In 2014, the European Food Safety Authority published a scientific opinion on PEDV and PDCoV summarizing the global epidemiological situation based on a review of the scientific literature published in the preceding 10 years (2004–June 2014) (29). In regards to transmission of these diseases in feed and non-animal origin feed ingredients, the European Food Safety Authority concluded that the scientific literature supports the following statements (29):

- High levels of infectious PEDV are shed in feces, contributing to contamination of various fomites, including vehicles, humans, and feed.
- The transmission of PEDV via feed has been shown but more data are required to assess its epidemiologic importance.
- Food waste fed to pigs (swill) can contain PEDV but epidemiologic role of this finding is unknown.

In 2016, the European Food Safety Authority published a scientific report of PEDV epidemiology and impact as reported in the scientific literature in 2014 and 2015, together with an analysis of PED cases in the European Union (30). Transmission of PEDV via feed or feed ingredients was not directly addressed in this report; however, the major recommendation(s) relevant to feed and feed ingredients included the importance of strict biosecurity, in particular with transport vehicles, to prevent introduction of PEDV onto the farm (30).

Although PEDV was first identified in Japan in the 1990s, Japan experienced renewed outbreaks of the disease in 2013. In a retrospective questionnaire-based case-control study, Sasaki et al. (26) focused on risk factors associated with outbreaks of PEDV in Japan on locally exposed farms and non-locally exposed farms. Feed-related items included on the questionnaire included frequency of feed truck visits and feeding of artificial milk (with or without SDPP). On multivariable analysis, no significant association was found between PED and artificial milk. PED was associated with increased feed truck visits to non-locally exposed farms but not locally exposed farms, suggesting transmission mechanisms differ between farms close to infected PED farms compared to farms further away (26).

In a report by Davies (4), the author discussed the similarities of 3 major disease epidemics in the swine industry caused by PRRSV, PCV2, and PEDV. The author points out that all three share the following common features: highly host specific, rapid rates of mutation, and appear to be associated with swine as non-pathogenic (or associated with mild disease) for years before becoming highly pathogenic. The author suggests that the pathogenic forms of these viruses evolved out of the existing swine virome^2^, arguing that intensive single species food production systems along with globalization, intensification of production practices, and multiple movements of pigs within the production cycle have contributed to the geographic expansion of these viral agents and points to the likelihood that future emerging or re-emerging viruses in swine are likely to arise from already recognized (non-pathogenic or ignored) swine viruses.

The author also discussed the risk of feeding animal products to swine and whether or not the risk warrants excluding animal origin products (or other ingredients) from swine diets. The author argues that complete bans on certain ingredients in swine feed may not be the solution. Rather, he suggests that one must take into consideration all contributing disease transmission pathways, the cost-benefits of mitigating risks associated with feed and feed ingredients, and the nutritional needs of commercial swine (4).

In a review by Lowe (24), the author summarized aspects of the U.S. outbreak of PEDV in 2013, including factors that were found to be associated with greater risk of PEDV transmission. The author concluded that current research supports transmission of PEDV through livestock transportation, movement of people, vehicles, and other contaminated fomites as well as shared resources and equipment among farms. Further, the author stated that feed was not likely a primary transmission route of PEDV in 2013 (24); however, the author cited other clinical studies and epidemiological investigations that provide evidence that feed can serve as a fomite for PEDV if contaminated during the manufacturing, storage, and transport processes (24).

In 2014, the United States experienced the emergence of a second novel swine enteric coronavirus, PDCoV. McCluskey et al. (25) administered a retrospective survey to 42 U.S. swine breeding herd operations that experienced a confirmed outbreak of PDCoV in order to identify factors that may have contributed to the introduction and spread of the virus. Among other things, the source and timing of feed delivered to the affected farms in the 10 days prior to the outbreak of PDCoV were examined. All farms surveyed had a delivery of complete feed or feed ingredients in the 10 days prior to the outbreak. One third of the farms received feed components from outside of the United States. There was no common source of feed or feed ingredients for the farms surveyed (25).

Scott et al. (27) formed a “Root Cause Investigation Group” to address the question of how PEDV entered the United States in spring 2013. The group used various methods including scenario development, *post-hoc* investigations, epidemiologic surveys, a case-control study, brainstorming, and speculation. They used previously collected epidemiologic data to develop scenarios and identify hypothetical routes of PEDV introduction into the United States and conducted follow-up studies to gather evidence for the most plausible scenarios. The follow-up studies included testing organic soybeans and pet jerky treats imported from China and archived serum samples opportunistically collected from feral swine; all results were negative. The authors did not identify a proven source or route of PEDV introduction into the United States. However, it was suggested that incomplete farm feed/ration records negatively impacted investigators' ability to thoroughly evaluate the potential epidemiologic role of feed or feed ingredients. The authors identified totes used to transport bulk feed or feed ingredients as providing “the simplest explanation” for the investigation findings (27).

Niederwerder and Hesse (28) administered a retrospective survey to U.S. swine veterinarians and producers in 2017 to collect opinions regarding, among other things, the suspected source of PEDV and PDCoV introduction into the United States in 2013–2014. At the time of the survey, the majority of respondents had either never experienced an outbreak of PED or PDCoV infection (28.9%) or reported that the virus had been eliminated from their farms (56.6%). The majority of respondents believed feed (29%), trucks coming onto the farm (26%), and variable biosecurity protocols (18%) were responsible for virus introduction. Survey participants were also asked about control measures implemented in response to outbreaks. None of the participants noted a change in feed practices although 56% implemented enhanced biosecurity protocols (28).

#### Feed and Feed Ingredients

Feed and feed ingredients have been hypothesized to serve as fomites for virus introduction and spread, leading several investigators to examine environmental samples (feed, fomites etc.) for the presence of virus and/or to conduct assays testing the ability of feed to serve as a fomite for virus. The following summarizes the reported findings.

Associated with the U.S. outbreak of PED in 2013, an epidemiological investigation was conducted at an affected Ohio swine operation to determine the source of virus introduction (31). The timing of the outbreak coincided with a switch to a new out-sourced feed pellet. Environmental samples were obtained and analyzed by real-time reverse transcription-polymerase chain reaction (RT-PCR). The investigators reported that PEDV RNA was detected in newly opened bags of pellets on-farm and in pellets and individual ingredients sampled at the source (supplier) facility. No virus isolation (VI) assays were performed on these samples. In the bioassay conducted, pigs were observed to be healthy and no clinical signs of disease were observed (31).

A retrospective case study by Greiner (32) investigated the presence of either PEDV or PDCoV RNA in select locations of commercial feed mills following the outbreaks of PED and PDCoV infections in the United States. To investigate the role, if any, of feed and feed mills in these outbreaks, environmental sampling was conducted at 24 feed mills, some of which served farms known to be positive for PEDV or PDCoV. The investigators swabbed office floors, bulk ingredient pit grates (exterior surfaces), mixer/pellet coolers, incoming bagged-ingredient truck trailers, the interior of feed compartments on trucks servicing farms, and feed truck foot pedals. None of the samples obtained were positive for PEDV RNA, 5% of truck foot pedals and 1% of bulk ingredient pit grates were suspect for PDCoV RNA, and 3.4 and 2.2% of truck foot pedals and office floors, respectively, were positive for PDCoV. All other samples were negative for PDCoV. With the exception of the 3.4% of suspect samples, none of the incoming ingredient trucks, bulk ingredient pits, or outgoing feed compartments were positive for PEDV or PDCoV RNA. There were no significant associations between viral RNA at feed mills and the disease status of farms served (32).

Trudeau et al. (33, 34) conducted experimental studies to investigate inactivation kinetics of PEDV, PDCoV, and TGEV in feed and feed ingredient matrices and on solid surfaces. Feed and non-animal origin feed ingredients used in these studies included complete feed, corn, soybean meal, corn dried distillers grains with solubles, and vitamin and trace mineral premix. The authors mixed stock virus with liquid medium, and then spiked samples of feed and feed ingredients with the mixture and incubated the combinations at various temperatures. The authors used a cell culture based assay and model fitting to estimate a delta value, calculated as an indicator of the time necessary to reduce virus concentration by 1 log. For the spiked samples incubated at room temperature, the greatest delta values were obtained for PDCoV and TGEV in soybean meal, at ~42 days each. Soybean meal at room temperature also provided the highest delta value for PEDV, at 7.5 days. Other findings indicated that at room temperature, moisture and ether content were important determinants of virus survival. The authors found no difference in virus survival in feed and non-animal origin feed ingredients incubated at temperatures higher than 70°C. The maximum level of virus inactivation occurred upon heating the spiked samples at 90°C for 30 min (33, 34).

#### Summary of Studies Regarding Epidemiology and Outbreak Investigations

In summary, the epidemiologic investigations and outbreak studies did not definitively link or exclude transmission of PEDV or PDCoV with non-animal origin feed or feed ingredients. Inconsistent findings impart uncertainty toward feed or feed ingredients as a transmission pathway (24, 25, 31, 32, 35). The most common mechanical fomite implicated in this group of studies was transport vehicles, including a positive association between feed truck movements onto farms and disease status (24–26, 28, 29, 32, 35).

Two studies conducted environmental sampling of feed, feed facilities, and feed transport vehicles to determine whether these items contributed to the cause of the outbreaks of PED and PDCoV infection in the United States (31, 32). Collectively, the findings indicate that none of the source ingredients nor the outgoing feed at the feed mills sampled were positive for viral RNA, suggesting the non-animal origin ingredients and feed were not contaminated with PEDV or PDCoV. However, experimental studies by Trudeau et al. (33, 34) did show that feed ingredients, soybean meal in particular, spiked with PEDV, PDCoV, and TGEV in a laboratory setting could maintain live virus for a period of time at room temperature. The authors also found that different ingredients supported different virus survival periods which they believe may be due to differences in moisture content, ether content and/or pH of each ingredient. These data indicate that, under certain conditions, swine enteric coronaviruses are able to survive in feed ingredients (33, 34). However, these studies did not investigate nor were they able to demonstrate that virus present in feed could be transmitted to naïve animals through normal feeding behavior.

Among the knowledge gaps identified in these studies are the identification of critical control points for pathogen contamination of feed and/or non-animal origin feed ingredients and consensus on the feed-based transmission pathways for PEDV and PDCoV. The potential point source(s) of virus contamination of non-animal origin feed or feed ingredients have not been identified nor clearly defined.

### Experimental Studies on Feed Transmission With Swine Bioassays

The rapid spread of PED in commercial swine in North America in 2013–2014 prompted several non-randomized, experimental studies with *in vivo* biological assays (bioassays) to be conducted. The bioassays were used to assess the biological activity or potency of the pathogen(s) of interest by measuring the magnitude of response such as observed clinical signs consistent with the study disease(s) and/or positive findings to diagnostic testing and necropsy examination. Most studies included in this section of the literature review used swine bioassays to determine the infectivity of the pathogen(s) of interest in feed or feed ingredients subsequent to detection by polymerase chain reaction (PCR) of virus nucleic acid in the matrix (feed or feed ingredients) under examination.

For the purposes of discussion, the experimental studies with swine bioassays were collated into two categories: field-based experimental studies with bioassays (11, 31, 36), and laboratory-controlled experimental studies with bioassays (37–41). In general, the bioassays involved naïve piglets of various ages, ranging from 4 to 21 days old (11, 41); in some studies, pigs were re-used after a negative bioassay and the subsequent ages at re-introduction to the bioassay were not easily discernable (38, 39). Piglets were sourced from healthy herds and tested by PCR and serological assays to confirm negative status for the respective pathogen(s) prior to the initiation of the bioassay. Exposure routes of the challenge matrix to the piglets varied, including *ad libitum* (natural) feeding (11, 31), oral administration via syringe (38, 39, 41), orogastric gavage (36, 37, 40), intramuscular injection, and intranasal administration (41). In most studies, the bioassays consisted of daily diagnostic monitoring, lasting 6–7 days post-inoculation, culminating with euthanasia and complete necropsy examinations of piglets.

#### Experimental Studies With Swine Bioassays Using Field-Sourced Challenge Virus

Three experimental studies with swine bioassays exposed piglets to challenge feed samples inoculated with field-sourced virus from an index farm or contaminated feed facility. Prior to the bioassay, the virus material was further processed or prepared in the laboratory before the piglets were inoculated with the challenge matrix. The bioassay results yielded mixed findings (11, 31, 36).

Pillatzki et al. (36) used a swine bioassay to investigate whether PEDV PCR-positive samples of complete feed, feed pre-mix, and SDPP (Ct values of 33.8, 34.2, and 30.0, respectively) that had been retained by feed manufacturers could serve as a source of PEDV transmission to neonatal swine. The piglets inoculated with the PEDV-positive feed samples along with the negative-control piglets remained negative for PEDV by PCR and clinically healthy throughout the study period. In contrast, only the positive-control piglets (Ct value = 25.5) developed clinical signs of PED. The authors provide several plausible explanations for the failure to demonstrate infectivity, including the nucleic acid detected in the feed samples did not represent infectious virus; the feed samples had relatively high Ct values (range: 30.0–36.5); and an extended storage time between collection of the sample and the bioassay may have reduced or eliminated the infectivity of the PEDV. Despite mixed results, Pillatzki et al. (36) concluded that feed contaminated with infectious PEDV could serve as a vehicle for disease transmission, citing as evidence that the positive-control piglets that were administered spiked feed did develop clinical signs of PED and PEDV fecal shedding.

Bowman et al. (31) conducted a swine bioassay on a newly started pelleted diet that was implicated as the transmission vehicle of PEDV into an Ohio swine operation. PEDV was detected by RT-PCR in the interior of the unopened bags of the new supplier's pellets, suggesting contamination occurred prior to delivery of the feed to the farm. Additionally, the source facility (supplier) tested positive for PEDV as did individual ingredients at the source facility. Piglets were provided *ad libitum* access to the RT-PCR positive mash^3^ (mean Ct = 36.5) along with dry pellets from the same lot for 7 days and observed for clinical signs of PED. During the bioassay, none of the pigs developed clinical signs of disease and diagnostic tests were negative for PEDV (31). Explanations for the failure to demonstrate infectivity were similar to those provided by Pillatzki et al. (36), with the addition that the small number of piglets used in the bioassay and the short feeding trial period lowered the sensitivity of the bioassay and did not realistically reflect the field setting. Despite the negative findings, Bowman et al. (31) stated “feed cannot be ruled out” as the source of the outbreak in the Ohio swine operation.

Following an outbreak of PED on three breeding herd premises in the United States, Dee et al. (11) used a novel on-farm sampling method to collect remnants of feed samples from empty feed bins that previously contained feed consumed by the index populations. Analysis of feed material across the 3 affected sites by real-time RT-PCR indicated the presence of PEDV RNA with Ct values ranging from 19.50 to 22.20. For the swine bioassay, piglets were divided into three groups: the treatment group; the positive-control group; and the negative-control group. Piglets were fed via natural feeding method. Clinical signs consistent with PED were observed in piglets in the treatment group and the positive-control group. At necropsy, rectal swabs and intestinal tract samples from the treatment group and the positive-control group were positive for PEDV by PCR and immunohistochemistry with evidence of microscopic lesions. In the negative group, clinical signs, viral shedding, or PEDV-positive intestinal tract samples were not observed. Molecular sequencing of viral RNA obtained from treatment and positive control groups confirmed consumption of feed and not cross-contamination as the source of infection (11).

#### Experimental Studies With Swine Bioassays Using Laboratory-Sourced Challenge Virus

Five experimental studies with swine bioassays involved laboratory-controlled experimental studies in which piglets were exposed to the challenge matrix spiked with a predetermined pathogen dose sourced from laboratory stock viruses (37–41). Goyal (37) and Schumacher et al. (40) conducted experimental studies with a swine bioassay aimed at determining PEDV survivability in various organic materials and minimum infectious dose, respectively. Three experimental studies with swine bioassays reported by the same primary author examined the infectivity of PEDV in common swine feed ingredients in the presence or absence of a formaldehyde-based liquid antimicrobial, SalCURB® (LA) (38); the infectivity of PEDV in common swine feed ingredients with or without LA and 2% custom medium chain fatty acid blend (MCFA) following a simulated trans-Pacific shipment from China to the United States (39); and the infectivity of select viral pathogens in common swine feed ingredients following simulated transportation conditions across two different regions of the world (41).

Goyal (37) investigated the survival of PEDV and TGEV in fresh feces, manure slurry, animal feed, and water. Stock PEDV were inoculated into samples of fresh feces, slurry, dry and wet swine feed, and drinking water and the mixtures were incubated at various humidity percentages and temperatures and for up to 14 days. PEDV and TGEV could be detected by PCR in fresh feces for 1–7 or 14 days, respectively, depending on temperature and humidity. Both viruses could be detected in slurry, non-chlorinated water, and dry and wet feed samples for ≥28 days at room temperature (37). From the bioassay results, this study suggests that PEDV inoculated into wet and dry swine feed can remain infective for up to 28 days and 7 days, respectively (37).

Schumacher et al. (40) used a swine bioassay to estimate the minimum infectious dose of PEDV in virus-inoculated feed. The authors mixed serial dilutions of stock PEDV with feed, and administered the mixtures to 10-day-old piglets by orogastric gavage. The feed used in the study was corn- and soybean meal-based and included vitamin and trace mineral premixes and a source of phytase. The lowest concentration of virus in feed to cause infection in the piglets was 5.6 × 10^1^ TCID_50_/g which corresponds to a Ct value of 37. Based on this infective dose, the authors estimated that 1 g of fecal matter could contaminate up to 450,000 kg of feed (40).

Dee et al. (38) examined PEDV viability in various feed ingredients common in swine diets in the presence or absence of LA. Eighteen common swine feed ingredients were selected: corn, conventional soybean meal (SBM), dried distillers grain with solubles (DDGS), SDPP, purified plasma, intestinal mucosa, meat and bone meal, red blood cells, 3 vitamin/trace mineral mixes, choice white grease, soy oil, lysine HCL, D/L methionine, threonine, limestone, and dry choline chloride. Samples of each ingredient were divided into two groups (treated and non-treated), in replicate, and spiked with PEDV. The samples were stored outside in winter conditions in plastic totes. At 1, 7, 14, and 30 days post-inoculation, samples were removed for diagnostic testing. The samples were tested for PEDV, PDCoV, and TGEV by RT-PCR. Viable PEDV, indicated by positive VI, at 30 days post-inoculation was detected from non-treated SBM, DDGS, red blood cells, meat and bone meal, lysine HCL, and D/L methionine. Non-treated choice white grease, threonine, and limestone were positive on VI at varying sampling days. Only SBM and meat and bone meal remained PCR positive at day 30; all LA-treated ingredients were VI negative (38).

The swine bioassay was used for PCR positive but VI negative feed samples, including the non-treated ingredients of corn, 3 vitamin/trace mineral mixes, intestinal mucosa, soy oil, choline chloride, SDPP, purified plasma, as well as the LA-treated ingredients. Choice white grease, limestone, and threonine were tested as well. Following completion of the bioassay, viable PEDV was detected only in piglets challenged with non-treated choline chloride and choice white grease (38).

Dee et al. (39) designed a model to evaluate the transboundary risk of PEDV-contaminated swine feed ingredients during a simulated shipment from China to the United States and tested the effect of two mitigation strategies aimed at decreasing the level of infectious PEDV in feed using LA and 2% custom MCFA. Fourteen swine feed ingredients commonly imported from China to the United States were selected: organic and conventional soybeans and soybean meal (SBM), lysine HCL, D/L methionine, tryptophan, vitamins A, D, and E, choline chloride, rice hulls, corn cobs, and feed-grade tetracycline. Each ingredient sample was organized into four batches, in replicate, each representing a specific segment of the 37 day shipping journey. In addition to positive and negative controls, samples were treated with LA or MCFA and spiked with PEDV. The samples were housed in a programmed environmental chamber based on the temperature and percent relative humidity for each segment of the shipping journey. At designated days post-inoculation, samples were removed and submitted for diagnostic testing by RT-PCR and VI. Viable PEDV, confirmed by VI, in the treatment batch representing shipment to and storage in Iowa was found in non-treated organic and conventional SBM, lysine, and vitamin D (39).

The swine bioassay was used to test ingredients that were PCR-positive for PEDV but negative by VI. This included non-treated ingredients vitamins A and E, tryptophan, D/L methionine, organic and conventional soybeans and choline chloride. Ingredients treated with LA or MCFA included organic and conventional soybean meal, lysine, vitamin D, and choline chloride. None of the piglets fed LA- or MCFA-treated ingredients spiked with PEDV were positive on the swine bioassay. Positive bioassay findings were observed in piglets that were administered non-treated choline chloride (39).

Dee et al. (41) evaluated the survival of select viral pathogens in feed ingredients using models designed to simulate transportation conditions across two different regions of the world. Eleven viruses were selected: foot-and-mouth disease virus, classical swine fever virus, ASFV, influenza A virus of swine, PRV, Nipah virus, PRRSV, swine vesicular disease virus, vesicular stomatitis virus, PCV2, and vesicular exanthema of swine virus. Surrogate viruses were used for foot-and-mouth disease virus, classical swine fever virus, PRV, vesicular exanthema of swine virus, Nipah virus, and swine vesicular disease virus. Eleven feed ingredients were selected: organic and conventional SBM, soy oil cake, DDGS, lysine HCL, vitamin D, choline chloride, moist cat food, moist dog food, dry dog food, and pork sausage casings. Two transboundary shipping journeys were modeled: trans-Pacific to simulate travel between China and the United States and trans-Atlantic to simulate travel between Europe (Poland) and the United States (for ASFV only). Similar methods to Dee et al. (39) were used for sample preparation, incubation, and diagnostic testing (LA or MCFA were not included). A wide variation in viability was observed across the virus-ingredient combinations (41).

The swine bioassay was used to determine infectivity of feed ingredients that tested positive by PCR but negative on VI in cell culture. The bioassay was performed with Senecavirus A (foot-and-mouth disease virus surrogate), PRRSV, porcine sapelovirus (swine vesicular disease virus surrogate), PCV2, ASFV, and influenza A virus of swine in selected virus-ingredient combinations. Pigs were inoculated by various methods, including orally via syringe, intramuscularly, and intranasally. A positive bioassay was observed for the following virus-ingredient combinations: PRRSV and conventional SBM; PRRSV and DDGS; Senecavirus A and choline; and PCV2 and lysine, choline, and vitamin D (41).

Additionally, the investigators reported that from the virus-ingredient combinations subjected to various simulated environmental conditions, 7 viruses remained in a viable form in 2 or more ingredients: Senecavirus A, ASFV, PRRSV, porcine sapelovirus, PCV2, feline calicivirus (vesicular exanthema of swine virus surrogate), and bovine herpesvirus-1 (PRV surrogate). The non-animal origin feed ingredients that supported survival of multiple viruses (*n*) included conventional SBM (*n* = 7), lysine (*n* = 5), vitamin D (*n* = 4), choline (*n* = 4), organic SBM (*n* = 3), and DDGS (*n* = 2). The findings suggest that viruses can survive in non-animal origin feed ingredients but survival duration is variable and dependent on virus properties and feed matrix (41).

#### Summary of Experimental Studies on Feed Transmission With Swine Bioassays

In summary, the infective dose of PEDV is low and, experimentally, infectivity of the feed material is dependent on viral load (e.g., Ct value) (31, 36, 37, 40). The findings in three studies, under different experiment conditions, suggest that virus survival is ingredient-dependent. Varying physical and chemical characteristics of feed ingredients may enhance or protect virus survival. The feed ingredients that have shown to support virus survivability and viability include conventional soybean meal, lysine, choline chloride, and vitamin D (38, 39, 41). Of interest, Dee et al. (41) noted that PED virus viability in organic SBM could not be demonstrated. This finding could discount previous speculation that the rise in organic swine farming may have contributed to PEDV introduction in to the United States. The authors also noted that ASFV demonstrated strong survivability characteristics, remaining viable under laboratory-simulated conditions with or without the feed matrix. Similarly, Senecavirus A, the surrogate for foot-and-mouth disease virus, showed the highest degree of stability as viable virus was recovered from 10 of 11 ingredients (41). Both formaldehyde-based LA and MCFA treatment rendered virus inactive, regardless of ingredient type, suggesting these mitigants might be useful as part of a risk management strategy for reducing viral load in feed ingredients (38, 39).

Among the knowledge gaps identified in these studies are the identification of vulnerable non-animal origin feed ingredients for viral contamination, ingredient (matrix) characteristics that support or hinder virus survival, and identification of the critical point(s) in the transboundary feed production and distribution continuum where viral contamination of non-animal origin ingredients could occur. There is a lack of field data demonstrating whether, how, and when non-animal origin feed ingredients may become contaminated with swine viruses. Further, although some field epidemiological investigations have associated contaminated (PCR positive) feed with the source of virus introduction on affected farms, to date, experimental studies of virus transmission via feed and feed ingredients have yielded inconsistent data. Additionally, the development of diagnostic assays and sampling techniques capable of detecting small amounts of virus in large volumes of non-animal origin feed ingredients is necessary to allow more accurate estimation of the frequency with which these materials are contaminated with virus, which viruses are present, and, if present, at what concentrations. Subsequent assays to determine the level of virus in the final, processed feed product would be useful to estimate the likelihood that contaminated non-animal origin feed ingredients could serve as a transmission pathway for swine viruses and to what degree feed should be prioritized as a biosecurity risk.

## Discussion

The objective of this literature review was to gather and analyze the evidence in published scientific literature regarding whether non-animal origin ingredients of commercial swine feed could introduce and transmit viral pathogens of swine into or within the United States. The goal was to understand the current scientific knowledge and to identify information gaps to better inform policy makers, swine industry stakeholders, and the scientific community. To achieve this, relevant literature was reviewed and findings were qualitatively summarized.

### Summary of Evidence in the Included Studies

The results of the literature review demonstrate that a limited number of studies currently address swine viral pathogen transmission through non-animal origin feed and feed ingredients. Despite the small number of published studies, there is relevant evidence in the literature that contributes to the on-going discussion regarding the potential role of non-animal origin feed ingredients in the transmission of swine pathogens, including:

- A subset of the studies reviewed provided experimental evidence that swine viruses can survive in non-animal origin feed ingredients under various experimental and laboratory conditions (21, 37–39, 41). Virus survival times were variable (ranging from 7 to >180 days) and dependent on the simulated environmental conditions applied (e.g., temperature and relative humidity) and the virus-ingredient combination.
- A subset of experimental studies provided evidence that feed contaminated with virus can transmit disease to naïve piglets (11, 37–41).
- Further, a subset of experimental studies attempted to identify individual feed ingredients that may be more likely than others to support virus survivability (38, 39, 41). The presence of a viable form (meaning a positive VI or bioassay) of virus at ≥30 days was confirmed in the following non-animal origin ingredients that were experimentally inoculated with virus (38, 39, 41):
  ◦ choline chloride
  ◦ D/L methionine
  ◦ dried distillers grain with solubles
  ◦ lysine HCL
  ◦ soybean meal
  ◦ vitamin D.

Extended survival of PEDV was observed in conventional SBM (38). Similarly, Dee et al. (41) found that feline calicivirus (surrogate for vesicular exanthema of swine virus) and Senecavirus A (surrogate for foot-and-mouth disease virus) had extended half-lives in conventional SBM.

- A subset of experimental studies concluded that duration of virus survival (and infectivity) is ingredient-dependent. Virus viability as determined in swine bioassay was observed with the following virus-ingredient combinations:
  ◦ PEDV-contaminated choline chloride (38, 39)
  ◦ Senecavirus A-contaminated choline chloride (41)
  ◦ PRRSV-contaminated conventional soybean meal (41)
  ◦ PRRSV-contaminated dried distillers grain with solubles (41)
  ◦ PCV2-contaminated choline chloride (41)
  ◦ PCV2-contaminated lysine HCL (41)
  ◦ PCV2-contaminated vitamin D (41)
- Under the laboratory-simulated model conditions, both LA and MCFA were concluded to be effective chemical mitigants against PEDV in individual feed ingredients stored under simulated shipping conditions, suggesting they might be useful for reducing viral load in feed ingredients (38, 39).

### Limitations of the Included Studies

Limitations in several studies hindered generalization to real-world commercial swine scenarios. For example, studies were limited by small feed sample volumes (as small as 5 grams) which does not directly equate to the quantities (tonnage) in actual swine production and feed scenarios; small sample sizes (2 replicates) which reduced statistical strength and confidence of findings; low sensitivity of swine bioassays (including low number of subject animals, *n* = 4); experimental methods which do not mimic natural feeding behaviors of swine or large-scale commercial swine production; and environmental scenarios which cannot be easily extrapolated to other seasonal variations or geographical regions.

Other studies, particularly those with retrospective questionnaire or survey components, were limited by inherent sources of internal bias such as selection and recall bias. Furthermore, robust replication of studies in independent laboratories and field settings to validate or corroborate findings has not occurred. Thus, conclusions drawn from these studies should be interpreted with caution until repeatability of the findings can be demonstrated, particularly under conditions that mimic the field setting.

### Summary of Knowledge Gaps in the Included Studies

While advancements in knowledge have been made, several areas warrant additional exploration and research. As mentioned above, evidence supports the conclusion that certain feed ingredients provide a more favorable matrix than others for extended survival (38, 39, 41). Several authors speculated that characteristics of the ingredient such as the physical supportive matrix and/or the chemical (bromatological) composition contributed to virus survival (21, 22, 38, 41). However, the specific characteristic(s) of the ingredient that contribute to viral persistence have not been identified. Additional research is needed to verify virus survival times (including half-life estimates) and infectivity in complete feed and feed ingredients, with various virus-ingredient combinations under various environmental conditions, including actual field conditions.

The outbreak epidemiological investigations provided evidence that the transmission route(s) for swine viruses onto the index farm may differ from the transmission route(s) among housing units within the index farm and between secondary farms. Similarly, the entry route for swine viruses into the United States may differ from the transmission route(s) among domestic swine farms. Thus, if swine viruses contaminate non-animal origin feed ingredients, a multi-modal transmission mechanism is likely to occur. When considering non-animal origin feed ingredients as potential fomites for swine virus transmission, it is important to understand how the primary transmission pathways (e.g., infected live pigs, contaminated transport vehicles, personnel) interface with one another, in particular, how the production and distribution of feed interacts with other potential or known sources of virus contamination (e.g., infected live pigs, contaminated transport vehicles, personnel) to contribute to disease transmission both into and within the United States. Similarly, it's important to understand the relative risks of various transmission pathways and where feed ingredients fit in among broadly accepted risk pathways such as movement of infected pigs and fecal contamination of fomites (e.g., transport vehicles). By understanding the magnitude of the risk of feed ingredients, one can better balance the costs of sourcing “safe” feed ingredients and the nutritional needs of pigs with the costs of applying various mitigation strategies to potentially contaminated feed (e.g., heat or chemical treatment or feed holding times).

Over the past several decades, the U.S. commercial swine industry has improved biosecurity measures on commercial premises to prevent transmission of economically significant viruses such as PCV2, PRRSV, and, most recently, PEDV. Commercial swine operations may use the absence of PRRS on the farm as an indicator of thorough implementation and enforcement of biosecurity measures. In the studies reviewed, Bowman et al. (31) stated that the “effectiveness of the biosecurity measures in place was evidenced by the absence of PRRS cases.” However, the entry of PEDV (and new or emerging swine viruses) onto presumably biosecure commercial premises suggests that current biosecurity standards may be insufficient to prevent certain virus incursions. Virus characteristics and the characteristics of the commercial swine industry, including globalization of trade, intensification and vertical integration of production, and extensive movement of pigs and related production components could contribute to biosecurity breaches. Robust biosecurity measures may be the only tool, in the absence of effective vaccines or treatments, to prevent the entry and spread of some diseases. Thus, biosecurity strategies, particularly the extensive movement of production inputs, need to be re-evaluated and adjusted to meet today's swine industry paradigm.

Arguably, the most crucial pieces of missing information are that neither the contamination routes of swine viruses into non-animal origin ingredients of commercial swine feed, leading to virus transmission to swine, nor the pathways of entry of exotic swine viruses into the United States have been definitively identified. A major knowledge gap exists in sources of potential contamination and where feed or feed ingredients may become cross-contaminated. Very little information is available on how non-animal origin feed ingredients are produced and sourced outside the United States and current studies have produced little scientific evidence of whether, how, or when non-animal origin feed ingredients become contaminated with swine viruses in regions outside the United States. The critical point(s) of susceptibility to contamination along the entire feed supply chain, from harvesting of plant-derived feed ingredients in the field to on-farm delivery of feed to swine premises, have not been identified. To compound this issue, reliable and validated assays and sampling techniques capable of detecting small amounts of virus in large volumes (bulk) of non-animal origin feed ingredients are not available. Taking a systematic approach to the entire (transboundary) feed production system, similar to the hazard analysis and critical control points process used in food safety, may help to identify vulnerabilities in the production process, better inform the development and application of mitigation measures to reduce viral contamination risks, and help stakeholders allocate resources toward mitigation measures based on the likelihood of virus contamination.

## Concluding Remarks

While investigators have addressed some critical questions pertaining to transmission of swine viruses via feed and feed ingredients, the current body of scientific knowledge has yet to provide conclusive evidence of virus contamination of non-animal origin feed ingredients with swine viruses and the epidemiology of virus transmission to swine via feed and feed ingredients under field conditions. If the primary concern lies in the importation of contaminated feed and feed ingredients, then additional research and investigative studies of how ingredients are sourced, processed, and transported prior to importation are needed. However, the lack of validated feed and feed ingredient diagnostic assays and sampling techniques capable of detecting small amounts of virus in large volumes of material limits the ability to determine whether and at what point non-animal origin feed or feed ingredients may become contaminated with viruses and limits the ability to identify critical control points in feed production, distribution, and storage to mitigate risk. Although LA and MCFA were shown to be effective chemical mitigants against PEDV, additional mitigation strategies should continue to be explored, including other chemical treatments, the application of heat or pressure (pelleting) to feed, and holding times for feed or feed ingredients. Moving forward, studies designed to examine the likely source(s) of contamination in the feed supply chain and virus mitigation steps in processing and post-processing may be the most fruitful focus of research.

Currently, the role of feed in the transmission of swine diseases is experiencing intense scrutiny and research from U.S. Federal and State government agencies, the swine industry, and the scientific community. The pool of knowledge is rapidly changing; thus, future consideration should be given to conducting an updated literature review to incorporate more recent findings (April 2018 to present).

## Author Contributions

RG, IK, and AM performed the literature searches. RG, KC, IK, DL, and AM reviewed the articles identified via the literature search, determined which articles met the inclusion criteria, and performed the data extraction and synthesis steps. RG, KC, IK, and DL authored the manuscript. All authors reviewed the manuscript, contributed to the design and research purpose statement of this review.

### Conflict of Interest Statement

The authors declare that the literature review was conducted in the absence of any commercial or financial relationships that could be construed as a potential conflict of interest. The views expressed in this article do not necessarily reflect the official policy of the U.S. Department of Agriculture or the U.S. Government.

## Footnotes

^1^World Organization of Animal Health (OIE) (2018). Available online at: http://www.oie.int/wahis_2/public/wahid.php/Diseaseinformation/WI/index/newlang/en (accessed November 16, 2018).

^2^The “swine virome” refers to the collection of nucleic acids (both RNA and DNA) that make up the viral community associated with swine species and its associated ecosystem.

^3^Mash refers to dry pellets, grain, or meal mixed with (hot) water to form a moist, pulpy feed.

## Abbreviations

ASF(V): African swine fever (virus)
Ct: cycle threshold
DDGS: dried distillers grain with solubles
LA: liquid antimicrobial
MCFA: medium chain fatty acid blend
PCV2: porcine circovirus type 2
PDCoV: porcine delta coronavirus
PED(V): porcine epidemic diarrhea (virus)
PHFD: porcine high fever disease
PRRS(V): porcine reproductive and respiratory syndrome (virus)
PRV: pseudorabies virus
SDPP: spray-dried porcine plasma
TGEV: transmissible gastroenteritis virus.
