# Supplementary material for: The Role of Non-animal Origin Feed Ingredients in Transmission of Viral Pathogens of Swine: A Review of Scientific Literature
Source: Front Vet Sci. 2019 Aug 22;6:273. doi: 10.3389/fvets.2019.00273 (PMC6714588; doi:10.3389/fvets.2019.00273)
Supplement: Supplementary file 1 [file Table_1.docx]

**Supplementary information**

**Table S1: Summary of data synthesis for the 26 included studies in the literature review**

| **Study** | **Study Design and Funding Source(s)** | **Viral Pathogen(s)** | **Fomite^^[[1]](#footnote-1)^^** | **Study Description** | **Core Outcomes** | **Methodological Comments** |
| --- | --- | --- | --- | --- | --- | --- |
| Bowman et al., 2015 | Epidemiological case report with bioassay  Funding provided by National Pork Checkoff, PIC North America, and USDA. | PEDV | Feed, starter feed pellets | Through epidemiological investigation of a swine operation in Ohio, contaminated feed (starter feed pellets) was identified as the likely source of PEDV introduction. The feed and feed source was RT-PCR-positive for PEDV. A bioassay was performed with samples of cryopreserved feed. | Authors determined the starter pellet to be the source of PEDV introduction. PEDV RNA was detected inside unopened bags of new pellets, at the source facility (supplier), and in individual ingredients at the source facility. For the bioassay, naive pigs fed PCR-positive feed from the supplier remained negative for PEDV. | The authors made several assumptions regarding biosecurity at the affected farm and ruled out other possible transmission routes without providing the supporting data. |
| Brookes et al., 2015 | Expert elicitation  Funding provided by Australian Pork Limited. | PRRSV | Multiple | Through expert elicitation, authors examine the most probable route of introduction of highly pathogenic PRRSV from southeast Asia into Australia. Participant answers were analyzed for commonalities and agreements were grouped and ordered. | Overall, significant agreement of respondents’ opinions for exposure routes involved disposal of waste to feral and backyard pigs. For commercial pigs, the highest probability exposure route was human fomite or access to animal feed/additives from SE Asia (very low). Less agreement on entry routes; animal feed/additives were given moderate probability. | The study design has several sources of potential bias, including participant selection, response/cognitive bias, framing bias, and recency bias. Authors examine multiple entry and exposure routes to analyze perceived risks by industry experts’ opinions. Thus, specific pathways (e.g. animal feed/additives) are not adequately defined or examined to the level of detail needed for in-depth analysis. |
| Davies, 2015 | Review article  Funding source not listed. | PRRSV, PCV2 and PEDV | Feed, SDPP | The article describes 3 major swine diseases, including PRRSV, PCV2 and PEDV. Similarities among the viruses are discussed. The virus transmission likelihood resulting from feeding animal products to swine and whether or not the true risk warrants excluding animal origin products from swine diets is examined. | The author suggests that "new" viruses in swine are likely to emerge from already recognized (non-pathogenic or ignored) swine viruses. The likelihood of PEDV survival in animal origin ingredients is extremely low, but not zero. A blanket ban on certain ingredients in swine feed may not be the solution. A comprehensive evaluation of transmission pathways as well as cost-benefit analyses of managing feed-related risks and nutritional value is needed. | The discussion focused on SDPP; however, key points can be generalized to non-animal origin ingredients. Increasing herd sizes (and assuming fixed biosecurity practices) along with greater flux of inputs translates to a higher temporal frequency of adverse events. The features of modern swine production (global trade, intense production, extensive movements) have contributed to the emergence of these pathogenic strains and we should expect this trend to continue. |
| Dee et al., 2014 | Experimental with bioassay  Funding provided by Pipestone Applied Research. | PEDV | Complete feed | At-risk feed bins were sampled on 3 index farms. Feed bins at 4 PEDV-negative farms were also sampled. All samples were tested for PEDV by RT-PCR. For the bioassay, 11 pigs were divided into 3 groups – a treatment group fed PCR-positive feed bin samples, a positive control group fed PEDV- spiked feed, and a negative control group fed a placebo. Groups were fed PCR-positive feed or placebo feed (ad libitum) on day 0 and PCR-negative feed throughout the remainder of the study. Pigs were necropsied on day 7 post- challenge. | Assessment of feed material in the at-risk bins across the 3 sites were PCR-positive for PEDV. All samples from control bins and PEDV-negative sites were PCR-negative.  For the bioassay, treatment and positive control groups exhibited clinical signs of PED and were PCR- positive for virus. The negative control group displayed no clinical signs of disease and were PCR-negative. | The bioassay sought to mimic on-farm transmission conditions by using PCR-positive feed material from confirmed PED affected farms and using a natural feeding method (ad libitum). An acknowledged limitation was that the *in vivo* study was not designed to estimate the frequency of feed-related PEDV infections. Results were based on a very small populations of pigs and cannot be extrapolated to today’s commercial farm and field conditions. |
| Dee et al., 2015 | Experimental with bioassay  Kemin Industries and Dr. Mark Bienhoff were acknowledged for providing technical expertise, funding and in-kind resources. | PEDV | Feed ingredients: corn, SBM, DDGS, SDPP, purified plasma, intestinal mucosa, MBM, RBC, 3 VTM mixes, white grease, soy oil, lysine HCL, D/L methionine, threonine, limestone, dry choline chloride | Common swine feed ingredients (18) were divided into two groups in replicate – LA treatment group and a non-treated group. Controls included complete feed spiked with PEDV (positive) or saline (negative). The samples were stored outside in winter conditions in plastic totes. At 1, 7, 14 and 30 DPI, feed samples were removed and were tested for PEDV, PDCoV, and TGE by RT-PCR. The presence of viable virus was tested by VI. A swine bioassay was conducted for PCR positive/VI negative feed samples. Piglets 5-7 days old were divided into groups of 4. Pigs received the designated inoculum orally via syringe and were observed for 7 days. The negative control group was given saline PO. If clinical signs observed, swabs were taken of diarrhea and vomit. Swabs were tested by PCR. If PEDV positive, all animals swabbed were euthanized; units were cleaned and piglets re-stocked as needed. | Only LA-treated samples of SBM and MBM remained PEDV-positive at 30 DPI. Supplementary testing of SBM (non-LA treated) was negative by PCR and VI for up to 210 days. PEDV was not detected in other treated ingredients. The following ingredients were used in the bioassay: (non- LA treated) corn, 3 VTM mixes, intestinal mucosa, soy oil, choline chloride, SDPP, purified plasma; (LA-treated) white grease, limestone, and threonine. Viable PEDV was detected only in piglets given choline and choice white grease.  PEDV viability may be influenced by ingredient type. Extended virus survival observed in SBM. LA is effective in rendering PEDV inactive, independent of ingredient type. | The study assumes post-processing contamination of ingredients; the index step or point of contamination in feed manufacturing/feed delivery is relatively unknown. Only 2 replicates per ingredient were used; confidence intervals and confidence levels were not provided. Small ingredient samples (30 g) may not equate to the quantities (tonnage) in actual swine production and feed scenarios. Outdoor winter weather conditions in this study may not be extrapolated to other climates or time periods. For the bioassay, pooled sampling was used across days 7, 14, and 30 DPI (not daily testing of samples) and piglets were re-used following negative bioassays. Bioassay inoculums were given orally via syringe which does not mimic normal feeding habits and may not correlate to field conditions. |
| Dee et al., 2016 | Experimental with bioassay  Kemin Industries, APC, Inc. and National Pork Board were acknowledged for providing technical expertise, funding and in-kind resources. | PEDV | Feed ingredients: organic and conventional soybeans, SBM, Lysine HCL, D/L methionine, tryptophan, Vitamin A, D, & E, choline chloride, rice hulls, corn cobs, and feed-grade tetracycline | The shipping journey from Beijing, China to Des Moines, Iowa was divided into 4 travel segments, represented by 4 sample batches. Two replicates of the 14 ingredients were allocated into the 4 travel segments. Each batch had a positive control group (PEDV with no LA treatment), LA-treated group and MCFA-treated group. Also negative controls (PEDV-negative feed with saline) and stock PEDV samples. Samples were housed in a programmed environmental chamber to mimic weather between China and Iowa in Dec 2012/Jan 2013. At designated DPI, samples were submitted for diagnostic testing with RT-PCR and VI. A bioassay was performed for PCR-positive, VI-negative samples. Piglets (5 days old) received inoculum (from batch 4) orally via syringe and were observed for 7 days. If clinical, swabs were taken of diarrhea and vomit and tested by PCR. If PCR-positive, animals swabbed were euthanized; units cleans and piglets re-stocked as needed. | First proof of concept study indicating PEDV survival in specific feed ingredients under modeled shipping conditions (China to US). After 37-day period, viable PEDV found (via VI) in Vitamin D, lysine hydrochloride, organic and conventional SBM. For the bioassay, piglets became PEDV-positive when administered non LA-treated choline chloride. Both LA and MCFA were concluded to be effective chemical mitigations as a means to reduce the risk of PEDV in feed ingredients. | The shipping timetable was based on one website, not (multiple) data points from actual shipping times and may not reflect real-life scenarios. Small ingredient samples (30 g) may not equate to the quantities (tonnage) in actual swine production and feed scenarios. Simulated environmental conditions may not be extrapolated to other environmental conditions. Bioassay inoculums were given orally via syringe which does not mimic normal feeding habits. In the discussion, authors may have overstated (or did not adequately provide justifications for) the “risk” of organic farming and imported soybean products. |
| Dee et al., 2018 | Experimental with bioassay  Funding provided by Swine Health Information Center (SHIC); American Association of Swine Veterinarians Foundation; State of Kansas National Bio and Agro-defense Facility Fund; SDSU Animal Disease Research and Diagnostic Laboratory. Salaries of four authors paid by Pipestone Applied Research. | SVA (FMDV)^^[[2]](#footnote-2)^^, bovine viral diarrhea virus (CSFV), BHV-1 (pseudorabies virus), canine distemper virus (Nipah virus), PSV (SVDV), FCV (vesicular exanthema of swine virus), ASFV, IAV-S, PRRSV, vesicular stomatitis virus, and PCV2. | Feed ingredients: organic & conventional SBM, soy oil cake, DDGS, lysine HCL, Vitamin D, choline chloride, moist cat food, moist dog food, dry dog food and pork sausage casings | Similar shipping model to Dee et al. 2016 employed. Two shipping routes: Trans-Atlantic (Poland to US) for ASFV and Tran-Pacific (China to US) for all other viruses. Eleven ingredient/11 virus combinations were assembled for trip segments. Five gram, gamma-irradiated samples were spiked with virus; stored in environmentally-controlled chambers. Each ingredient/virus combo was tested by RT-PCR and VI on the appropriate day (based on simulated travel). A bioassay was performed for PCR-positive, VI-negative samples, which included SVA, PRRSV, PSV, PCV2, ASFV, and IAV-S. | Seven of 11 viruses remained viable in 2 or more ingredients (SVA, ASFV, PRRSV, PSV, PCV2, FCV and BHV-1). SVA was recovered from 10 of 11 ingredients. ASFV samples survived the simulated 30-day shipping in the absence of feed matrix. FCV and SVA had extended half-lives in conventional SBM; SVA had the most stable half-life range (1.7 to 9.7 days across 10 ingredients). FCV had the longest survival in conventional SBM (26.6 days). Findings indicate viruses can survive in feed; survival duration is variable and dependent on virus properties and feed matrix. Data indicates non-enveloped viruses are more resistant in the environment than enveloped. | Small ingredient samples (5 g) may not equate to the quantities (tonnage) in actual swine production and feed scenarios. Confidence intervals were not calculated due to too few replications. Samples were spiked with the same amount of virus which may not reflect proposed field contamination. Bioassay inoculums were given IM, IN or orally via syringe which does not mimic normal feeding habits. Results seem to negate previous reports (Dee et al. 2016) that organic soybean products pose an increased “risk” of virus transmission compared to non-organic ingredients. |
| EFSA AHAW Panel, 2014 | Descriptive; qualitative literature review 2004-2014  Funding source not listed. | PEDV, PDCoV | N/A | At the request of the European Commission, EFSA AHAW Panel was tasked to deliver a scientific opinion on the current scientific evidence, epidemiological situation, and knowledge/data gaps regarding PEDV and PDCoV. | Transmission of these viruses in feed or feed ingredients was not directly addressed in this report. Overall, the major recommendation(s) relevant to NOFI included the importance of strict biosecurity, in particular with vehicles, to prevent introduction of PEDV onto the farm. | Comprehensive summation of the current knowledge of PEDV from 2004 to September 2014. |
| EFSA, 2016 | Descriptive; qualitative literature review Oct 2014 - Oct 2015  Funding source not listed. | PEDV | N/A | The European Commission requested EFSA to 1) provide guidance on PEDV data to be collected by EU Member States in order to optimize coordination of response, and 2) analyze the epidemiological data from EU Member States and in the scientific literature. The review focused on occurrence of infection with different PEDV strains, morbidity/mortality rates and severity of clinical disease. | Conclusions and recommendations starts pg. 20 & many annexes collate the findings and data from the updated literature review. | EU-centric report; however, recommendations pg. 20-21 are generalizable to US. For the impact of PEDV to EU farms, the authors noted in Table 3 that data were missing and analysis is difficult for non-reportable diseases. Due to the missing data, results in Table 3 must be interpreted with caution. |
| Fasina et al., 2012 | Retrospective case-control study  Funding source not listed. | ASFV | Feed (swill), water, rodents, equipment, people | A survey of farm characteristics, farm operations, and self-reported biosecurity measures was administered to case and control farm owners. Statistical analysis was performed on responses and risk factors using univariable and multivariable conditional logistic regression models. | Protection of feed and water (from rodents) and purchasing of commercial feed (vs swill feeding) was negatively associated (protective) with acquiring ASFV. Presence of abattoir in the community and infected neighboring farms was positively associated with risk for ASFV. | The study examined associative relationships between farm practices and risk of ASFV. Swill feeding was not defined but presumably swill would contain both animal and non-animal origin feed components. Self-reporting and recall bias is possible given study design. |
| Goyal, S.M., 2014 | Experimental; quasi-experimental  Funding provided by Pork Checkoff. | PEDV; TGE virus | Feces, slurry, wet and dry feed, water | PEDV and TGE virus were inoculated into animal feed (dry and wet) and water. Feces and slurry from infected animals were tested for virus. Spiked feed was incubated and instilled into the esophagus of piglets. Animals were scored for signs of PED/TGE and sacrificed. | PEDV and TGE virus were detected by RT-PCR in dry and wet feed, feces samples, slurry and water. All piglets inoculated with spiked feed became infected with PEDV at viral dilutions up to 10^-9^. | The study used a small sample set for the bioassay (12 piglets). No statistical analyses were performed for the experiments. Bioassay inoculation performed by gavage instead of ad libitum feed. Author demonstrates that virus can survive in various organic materials and that pooled samples of spiked feed can infect piglets with PEDV. Authors attempt to extrapolate findings to infections that occur in the field but this is unsubstantiated. |
| Greiner, L.L., 2016 | Descriptive  Funding provided by the National Pork Board. | PEDV; PDCoV | Feed mill fomites: office floors, bulk ingredient pit grates, trucks carrying bagged ingredients, mixer/pellet cooler, inside feed compartment on feed truck, foot pedals of feed delivery truck | Fomites at 24 US feed mills were swabbed for 5 consecutive days. Samples were analyzed by PCR at 1 of 4 laboratories. Eighteen of the 24 feed mills serviced farms known to be positive for PEDV; 5 delivered to PDCoV-positive farms. | No samples tested positive for PEDV RNA; 5% of truck foot pedals and 1% of bulk ingredient pits were suspect for PDCoV RNA; 3.4% of truck foot pedals and 2.2% of office floors were positive for PDCoV RNA. No bulk ingredient pits or mixer/coolers were positive for either virus. | The study examined various control/entry points for virus at feed mills. Authors did not find a positive correlation between virus presence at the feed mill and probability of PEDV infection on farms serviced. |
| Guinat et al., 2016 | Review Article  Funding provided by the European Seventh Framework Programme. | ASFV | Review examining ASFV transmission (pig-to-pig, fomites, ticks, feed) | Research summarized describes transmission of ASFV via feeding of contaminated animal meat. ASFV was not transmitted by contaminated sweet potatoes or bananas (1921 study). It was reported that a study in East Africa showed that ASFV was not transmitted by consuming non-animal origin feed (review 1969). | Authors conclude current research supports transmission of ASFV in feed containing contaminated swine products. Transmission through non-animal origin feed is less conclusive. Additional studies are required to determine if transmission occurs in feed not containing swine products. | In the section on feed, authors conclude viral strain may impact transmission via this route. In this respect, extrapolation of findings to other viruses/different feeds may not be appropriate. |
| Le et al.,  2012 | Retrospective survey  Funding source not listed. | PHFDV | N/A | Retrospective survey classified cases of PHFD in southern Vietnam. Statistical analysis was conducted to identify potential risk factors associated with disease status at household level. | The study found PHFDV prevalence was 33.4% and risk factors included: higher numbers of sows and finishing pigs, receiving pigs from an external source and the interaction between using ‘water green crop’ as pig feed and owning ducks with or without direct contact with pigs. | The smaller study area limits extrapolation of results to other areas. Case identification was based only on clinical signs and no diagnostic assays were used. Farm size is a confounding factor. |
| Lowe, J. F.  2014 | Review article  Funding source not listed. | PEDV | Feed, vehicles, people, and other fomites | The article reviews the US PED outbreak in 2013. The author summarizes clinical signs, virus shedding, immune responses, epidemiology, origin of the outbreak and risk factors contributing to transmission such as herd/farm management, transportation and fomites. | In this outbreak, PEDV was transmitted by livestock transport, movement of people, vehicles, and other contaminated fomites, and shared resources/equipment. The author suggests feed contaminated with infectious fecal material could transmit virus and that enhanced control procedures may provide protection against outbreaks of PED or other novel diseases in the future. | None |
| Martinez-Gamba et al.,  2001 | Experimental  Funding provided by PAPIIT-UNAM Project # INI210997. | Aujeszky's Disease virus; Blue Eye Disease virus | Ensilages (solid fraction of pig feces) | Swine feces was obtained from 30 pigs to prepare ensilage. A serological survey of the animals was performed to see if they were free of both pathogens. ADV and BEDV were inoculated into micro-silos and tested by viral identification methods. | No animals had antibodies against either the ADV or the BEDV and all samples obtained from micro-silos at different times of ensilage were negative for both viruses. Immunofluorescence and electron microscopy were positive only at 60 min after inoculation. | Due to the small sample set (1 farm, 30 animals, and 5-15 samples from each area) the study is not easily extrapolated to other farms/conditions. Ensilage appears to inactivate both viruses examined. |
| McCluskey et al.,  2016 | Retrospective testing; case series study  Funding source not listed. | PDCoV | Feed, feed components, trucks, equipment and drivers, farm employees, and visitors | Banked samples (feces, fecal swabs, intestines, or oral fluids) from commercial swine farms in 27 states were tested by PCR to estimate initial time point of PDCoV introduction. A survey was conducted to examine biosecurity practices and disease status over time. | Only 4 samples out of 2286 were PCR-positive for PDCoV. Nearly 29% of sites with ill gestating sows and gilts that purchased feed delivered in the 10 days prior to onset of PDCoV sourced feed ingredients from outside the US.  The authors conclude that the earliest detections in August and October 2013 may have had limited spread due to warm summer and fall temperatures. | The study examined a small number of operations (42 breeding farms). Authors did not discuss potential sources of bias – (e.g. survey and recall biases). |
| Niederwerder and Hesse,  2018 | Review article  Funding provided by the National Pork Board. | PEDV, PDCoV | Feed, trucks | Review examined SECV detection, epidemiology, and control efforts in the U.S. and Canada. Transmission and risk factors for introduction were also discussed. A survey of U.S. swine veterinarians and managers was conducted to compile information related to SECV including suspected sources of introduction. | The authors conclude that fecal–oral is the primary transmission route for SECV. Those surveyed (73.6%) believed truck movements onto farms, feed and biosecurity issues were the most likely routes of SECV introduction. | Survey results cannot be extrapolated due to the low number of respondents (40) and small number of herds (83). |
| Pillatzki et al., 2015 | Experimental with bioassay  Funding provided by the American Association of Swine Veterinarians. | PEDV | Complete feed, feed pre-mix, and dried porcine plasma retained by feed manufacturers from April and May 2013 | Investigators obtained 3 PEDV feed samples - complete feed, feed pre-mix and SPDD. After confirming the feed samples were PCR-positive, they performed a swine bioassay. Piglets were separated into 5 groups and inoculated with untreated feed (negative control), one of the PEDV-contaminated feeds (3 experimental groups), or feed spiked with PEDV stock virus (positive control). Feed samples were mixed with saline and supernatants were instilled into animals by gavage. Rectal swabs were collected daily. Pigs were euthanized on day 7, necropsied, and small intestine and colon samples were collected for analysis. | No clinical signs were observed in piglets from the negative controls or treatment groups inoculated with PCR-positive pre-mix feed, SDPP, or complete feed. Also, fecal swabs collected from these groups were PEDV-negative, no histologic lesions were, and PEDV was not detected by IHC. The positive control group developed clinical signs at 3 DPI, and feces was PCR-positive. No histology or IHC results were presented for positive controls. | The extended storage of the feed samples might have impacted virus viability. Authors suggest that these contaminated feed samples might not have been representative of the overall concentration of PEDV in the entire batch of feed. The method of inoculation for the bioassay (gastric gavage) does not reflect natural field transmission conditions. |
| Pirtle and Beran, 1996 | Experimental  Funding provided by Iowa Pork Producers. | PRRSV | Solid- stainless steel, plastic, boot rubber  Porous- ground corn, pelleted swine starter feed mix, wood shavings, alfalfa, straw, denim cloth  Liquid- PBSS, saline G, well water, city water, and swine urine, saliva, and fecal slurry | Three solid fomites, 6 porous fomites, and 7 liquids (25-27 °C) were contaminated with PRRSV. Samples were obtained on day 0 through day 11 for VI, cell culture assay, and fluorescent antibody staining. | Only the day 0 samples of the 3 solid fomites contained PRRSV. PRRSV was isolated only at day 0 for 3 porous fomites (alfalfa, wood shavings, and straw) and not detected in any samples for 3 porous fomites (corn, swine starter feed and denim cloth). PRRSV was isolated only at day 0 from all swine secretions (urine, saliva, fecal slurry). PRRSV was detected in two buffer solutions through days 4 and 6; in well water through day 9; and in city water through day 11. | Fomites were spiked with stock virus at doses that may not reflect contamination levels under field conditions, therefore, extrapolation to field conditions is limited. |
| Sasaki et al., 2016 | Retrospective case-control study  Funding provided by a KAKENHI Grants-in-Aid for Scientific Research) from the Japan Society for the Promotion of Science. | PEDV | People, trucks, equipment, feed, artificial milk, manure, pests | Japanese swine producers were surveyed for information regarding herd management practices for a two-week time period relevant to PEDV exposure. The goal of the study was to test the hypothesis that factors associated with high risk of PEDV infection were different for locally exposed farms (within 5 km of another PEDV-infected premises) than for non-locally exposed farms (greater than 5 km of another PEDV-infected farm). Investigators sought to quantify the dynamics of PEDV spread and support the design and implementation of PED prevention and control measures in Japan. | For locally-exposed farms, 8 of 20 variables were associated with PED status such as, increased farm size, shorter distances to the closest PEDV-positive farm, and a disinfectant contact time of less than 20 minutes. In non-locally exposed farms, PED status was associated with increased feed truck visits to the farm, visits by a veterinarian, and disinfectant contact time of less than 20 minutes. | Authors report that participants selected for this study may not be representative of the overall Japanese swine industry. Results should be interpreted cautiously. |
| Schoenbaum et al., 1991 | Experimental study  Funding provided by a grant from USDA APHIS. | PRV | Swine nasal washings, saliva, & urine, swine lagoon water and pit effluent, swine bile, chlorinated water, well water, heat-sterilized chlorinated water, heat-sterilized PBSS, steel, concrete, polypropylene plastic, vinyl rubber, denim cloth, loam soil, green grass, whole corn, pelleted feed (starter and finisher), MBM, alfalfa, straw, wood, swine feces | Fomites were spiked with stock virus, and the combinations were incubated at 25 °C. Samples were collected on days 0, 1, 2, 3, 4, 7, and 10, or until a PRV titer of < 10 PFU/ml was obtained. Swine bile was also sampled at 1 h, and swine urine on day 14. Virus titers were determined by counting plaques in cell culture. | Of the combinations of PRV and diluent with feed or non-animal origin feed ingredients, the combination of PRV/saline/whole corn remained infectious longest, at 7 days with an estimated half-life of 36.3 hours. The durations of infectiousness of the other combinations of PRV/diluent/feed or non-animal origin feed ingredients ranged from 1 to 4 days with an estimated half-life of 1.0 h to 5.1 h. Authors report that the “quantity of infectious virus decreased logarithmically” over time. The rate of decrease varied among fomites. | Generalizability of study findings to field conditions is poor. Non-animal origin feed and feed ingredients were mixed with diluents prior to spiking with virus. Field conditions detrimental to virus activity (drying and UV light exposure) were deliberately not used. |
| Schumacher et al., 2016 | Experimental with bioassay  Funding provided by the National Pork Board. | PEDV | Feed containing corn, SBM, VTM, and a source of phytase | Feed was mixed with stock PEDV at various doses, and the mixtures were administered to pigs by orogastric gavage. Fecal swab specimens were collected. Pigs were euthanized at 7 days after exposure. Fecal swab samples, tissue samples, and cecal contents were analyzed by PCR, histology, and/or immunohistochemistry. Virus titers were determined by RT- (quantitative) PCR. | The lowest concentration of virus in feed to cause infection in pigs was 5.6 x 10^1^ TCID_50_/g. The PCR cycle threshold value was 10 units lower for PEDV mixed with feed than for an equivalent dose of PEDV mixed with tissue culture medium. | Generalizability of study findings to field conditions is poor; virus-spiked feed was administered to pigs by orogastric gavage. |
| Scott et al., 2016 | Scenario development, post hoc investigation, epidemiologic survey, case/control, brainstorming and speculation  Funding source not listed. | PEDV, SECV | Products or equipment identified as having the potential to carry PEDV or other SECVs: feed totes, organic soybeans, pet treats, SDPP, biologicals, plant materials, amino acid supplements, and VTM | The authors used previously collected epidemiologic data to develop scenarios and identify possible routes of PEDV introduction into the United States, and initiated follow-up studies “to gather more evidence for the most plausible scenarios”. | No PEDV was detected in imported organic soybeans, pet jerky treats, or feral swine samples. Source of epidemic was not identified. Authors identified totes used to transport bulk feed as providing the simplest explanation for the investigation findings. | Unclear objective(s) and reporting. Appears to be an emphasis or assumption towards identifying Asia or China as the location of origin and towards imported organic soybeans as the point source feed ingredient. |
| Trudeau et al., 2017a | Experimental study  Funding provided by the National Pork Board. | PEDV, PDCoV, TGEV | Complete  feed, SDPP, meat meal, MBM, blood meal, corn, SBM, and DDGS. | Fomites were spiked with stock virus, and the combinations were incubated at room temperature for 0 to 56 days. Virus titers were determined through use of a cell-culture-based assay. | The first log decrease in PDCoV and TGEV activity took longest in SBM. Moisture and ether content were indicated as being important determinants of virus survival in feed ingredients. | Generalizability to field conditions is poor. Fomites were spiked with stock virus. Increased moisture content due to virus inoculation likely altered virus survival kinetics. |
| Trudeau et al., 2017b | Experimental study  Funding provided by the National Pork Board. | PEDV | SBM, swine growing-finishing  VTM, SDPP, meat meal, MBM, blood  meal, corn, and DDGS, complete feed, galvanized steel, stainless steel, aluminum, plastic | Feed and feed ingredients were spiked with stock virus, and the combinations were incubated at various temperatures for 0-30 min. Four fomite surfaces were spiked with stock virus and held at various temperatures for 0-10 days. Virus titers were determined through use of a cell-culture-based assay. | The authors found no difference in virus survival in feed or feed ingredients at temperatures higher than 70 °C. Maximum virus decrease occurred upon heating at 90 °C for 30 min. Inactivation kinetics did not differ among the surfaces tested. | Generalizability to field conditions is poor. Fomites were spiked with stock virus. Increased moisture content due to virus inoculation likely altered virus survival kinetics. |

**Abbreviations**

| ADV | Aujeszky’s disease virus | RBC | red blood cells |
| --- | --- | --- | --- |
| AHAW | Animal Health and Welfare | (RT)-PCR | (real time)-polymerase chain reaction |
| BEDV | blue eye disease virus | RNA | ribonucleic acid |
| Ct | cycle threshold | SBM | soybean meal |
| DDGS | distillers dried grains with solubles | SDPP | spray dried porcine plasma |
| DPI | day(s) post-inoculation | SECV | swine enteric coronavirus |
| EFSA | European Food Safety Authority | SVA | Seneca virus A |
| EU | European Union | SVDV | Swine vesicular disease virus |
| FCV | feline calicivirus | TCID_50_ | tissue culture infectious dose 50 |
| FMDV | foot and mouth disease virus | TGE(V) | transmissible gastroenteritis (virus) |
| IAV-S | influenza A virus of swine | VI | virus isolation |
| LA | liquid antimicrobial | VTM | vitamin/trace mineral |
| MBM | meat and bone meal |  |  |
| MCFA | medium chain fatty acid |  |  |
| PBSS | phosphate buffered saline solution |  |  |
| PCV2 | porcine circovirus 2 |  |  |
| PDCoV | porcine deltacoronavirus |  |  |
| PED(V) | porcine epidemic diarrhea (virus) |  |  |
| PHFDV | porcine high fever disease virus |  |  |
| PRV | pseudorabies virus |  |  |
| PSV | porcine sapelovirus |  |  |
| RBC | red blood cells |  |  |
| (RT)-PCR | (real time)-polymerase chain reaction |  |  |
| RNA | ribonucleic acid |  |  |
| SBM | soybean meal |  |  |

References

Bowman, A.S., Krogwold, R.A., Price, T., Davis, M., and Moeller, S.J. (2015). Investigating the introduction of porcine epidemic diarrhea virus into an Ohio swine operation. BMC veterinary research 2015 v.11 no.1, pp. 38-38. DOI: 10.1186/s12917-015-0348-2.

Brookes, V.J., Hernandez-Jover, M., Holyoake, P., and Ward, M.P. (2015). Industry opinion on the likely routes of introduction of highly pathogenic porcine reproductive and respiratory syndrome into Australia from south-east Asia. Aust Vet J 93, 13-19. DOI: 10.1111/avj.12284.

Davies, P.R. (2015). The dilemma of rare events: Porcine epidemic diarrhea virus in North America. Prev Vet Med 122, 235-241. DOI: 10.1016/j.prevetmed.2015.08.006.

Dee, S., Clement, T., Schelkopf, A., Nerem, J., Knudsen, D., Christopher-Hennings, J., et al. (2014). An evaluation of contaminated complete feed as a vehicle for porcine epidemic diarrhea virus infection of naïve pigs following consumption via natural feeding behavior: proof of concept. Bmc veterinary research 10.

Dee, S., Neill, C., Clement, T., Singrey, A., Christopher-Hennings, J., and Nelson, E. (2015). An evaluation of porcine epidemic diarrhea virus survival in individual feed ingredients in the presence or absence of a liquid antimicrobial. Porcine health management 1, 9. DOI: 10.1186/s40813-015-0003-0.

Dee, S., Neill, C., Singrey, A., Clement, T., Cochrane, R., Jones, C., et al. (2016). Modeling the transboundary risk of feed ingredients contaminated with porcine epidemic diarrhea virus. Bmc veterinary research 12, 51. DOI: 10.1186/s12917-016-0674-z.

Dee, S.A., Bauermann, F.V., Niederwerder, M.C., Singrey, A., Clement, T., de Lima, M., et al. (2018). Survival of viral pathogens in animal feed ingredients under transboundary shipping models. PLoS One 13, e0194509. DOI: 10.1371/journal.pone.0194509.

EFSA (European Food Safety Authority) (2016). Scientific report on the collection and review of updated epidemiological data on porcine epidemic diarrhoea. 14(2), 4375. DOI: 10.2903/j.efsa.2016.4375.

EFSA AHAW Panel (EFSA Panel on Animal Health and Welfare) (2014). Scientific Opinion on porcine epidemic diarrhoea and emerging porcine deltacoronavirus. EFSA Journal 12(10), 3877. DOI: 10.2903/j.efsa.2014.3877.

Fasina, F.O., Agbaje, M., Ajani, F.L., Talabi, O.A., Lazarus, D.D., Gallardo, C., et al. (2012). Risk factors for farm-level African swine fever infection in major pig-producing areas in Nigeria, 1997-2011. Prev Vet Med 107, 65-75. DOI: 10.1016/j.prevetmed.2012.05.011.

Goyal, S.M. (2014). Environmental stability of PEDV (porcine epidemic diarrhea virus). Research Report Swine Health.

Greiner, L.L. (2016). Evaluation of the likelihood of detection of porcine epidemic diarrhea virus or porcine delta coronavirus ribonucleic acid in areas within feed mills. Journal of swine health and production 24, 198-204.

Guinat, C., Gogin, A., Blome, S., Keil, G., Pollin, R., Pfeiffer, D.U., et al. (2016). Transmission routes of African swine fever virus to domestic pigs: current knowledge and future research directions. Veterinary record 178, 262-267. DOI: 10.1136/vr.103593.

Le, H., Poljak, Z., Deardon, R., and Dewey, C.E. (2012). Clustering of and risk factors for the porcine high fever disease in a region of Vietnam. Transbound Emerg Dis 59, 49-61. DOI: 10.1111/j.1865-1682.2011.01239.x.

Lowe, J.F. (2014). Porcine epidemic diarrhoea virus in the USA: lessons learned from the 2013 outbreak. Cab reviews 9, 1-4. DOI: 10.1079/PAVSNNR20149042.

Martinez-Gamba, R., P., P.-R., P., C.F., M., H., E., G., and C., M. (2001). Persistence of Escherichia coli, Salmonella choleraesuis, Aujeszky's Disease virus and Blue Eye Disease virus in ensilages based on the solid fraction of pig faeces. Journal of applied microbiology 91, 750-758.

McCluskey, B.J., Haley, C., Rovira, A., Main, R., Zhang, Y., and Barder, S. (2016). Retrospective testing and case series study of porcine delta coronavirus in U.S. swine herds. Preventive veterinary medicine 2016 v.123, pp. 185-191. DOI: 10.1016/j.prevetmed.2015.10.018.

Niederwerder, M.C., and Hesse, R.A. (2018). Swine enteric coronavirus disease: A review of 4 years with porcine epidemic diarrhoea virus and porcine deltacoronavirus in the United States and Canada. Transboundary and emerging diseases.

Pillatzki, A.E., Gauger, P.C., Madson, D.M., Burrough, E.R., Zhang, J., Chen, Q., et al. (2015). Experimental inoculation of neonatal piglets with feed naturally contaminated with porcine epidemic diarrhea virus. Journal of swine health and production 23, 317-320.

Pirtle, E.C., and Beran, G.W. (1996). Stability of porcine reproductive and respiratory syndrome virus in the presence of fomites commonly found on farms. J Am Vet Med Assoc 208, 390-392.

Sasaki, Y., Alvarez, J., Sekiguchi, S., Sueyoshi, M., Otake, S., and Perez, A. (2016). Epidemiological factors associated to spread of porcine epidemic diarrhea in Japan. Preventive veterinary medicine 123, 161-167. DOI: 10.1016/j.prevetmed.2015.11.002.

Schoenbaum, M.A., Freund, J.D., and Beran, G.W. (1991). Survival of pseudorabies virus in the presence of selected diluents and fomites. Journal of the american veterinary medical association 198, 1393-1397.

Schumacher, L.L., Woodworth, J.C., Jones, C.K., Chen, Q., Zhang, J., Gauger, P.C., et al. (2016). Evaluation of the minimum infectious dose of porcine epidemic diarrhea virus in virus-inoculated feed. Am J Vet Res 77, 1108-1113. DOI: 10.2460/ajvr.77.10.1108.

Scott, A., McCluskey, B., Brown-Reid, M., Grear, D., Pitcher, P., Ramos, G., et al. (2016). Porcine epidemic diarrhea virus introduction into the United States: root cause investigation. Preventive veterinary medicine 123, 192-201. DOI: 10.1016/j.prevetmed.2015.11.013.

Trudeau, M.P., Harsha, V., Urriola, P.E., Sampedro, F., Shurson, G.C., and Goyal, S.M. (2017b). Survival of Porcine Epidemic Diarrhea Virus (PEDV) in thermally treated feed ingredients and on surfaces. Porcine health management 3.

Trudeau, M.P., Verma, H., Sampedro, F., Urriola, P.E., Shurson, G.C., and Goyal, S.M. (2017a). Environmental persistence of porcine coronaviruses in feed and feed ingredients. Plos one 12. DOI: 10.1371/journal.pone.0178094.

1. A fomite is defined as an inanimate object or material that is likely to carry infection such as animal feed, feed ingredients, organic substrates, transport vehicles, boots, etc. [↑](#footnote-ref-1)
2. Surrogate viruses were used for viruses listed in parentheses. For other viruses listed, actual virus was used. [↑](#footnote-ref-2)
