# Supplementary material for: The Role of Non-animal Origin Feed Ingredients in Transmission of Viral Pathogens of Swine: A Review of Scientific Literature
Source: Front Vet Sci. 2019 Aug 22;6:273. doi: 10.3389/fvets.2019.00273 (PMC6714588; doi:10.3389/fvets.2019.00273)
Supplement: Supplementary file 2 [file Data_Sheet_1.PDF]

Data Sheet S1: Studies (n = 70) excluded after full text review

(2004). Studies on pathogens in feed. A conference report from Germany. Kraftfutter 87, 282-284.

(2015). Feed tote bags implicated in pig disease spread. J Am Vet Med Assoc 247, 1083-1084.

Alvarez, J., Goede, D., Morrison, R., and Perez, A. (2016). Spatial and temporal epidemiology of porcine epidemic diarrhea (PED) in the Midwest and Southeast regions of the United States. Preventive veterinary medicine 123, 155-160. DOI: 10.1016/j.prevetmed.2015.11.003.

Amass, S.F., Mason, P.W., Pacheco, J.M., Miller, C.A., Ramirez, A., Clark, L.K., et al. (2004). Procedures for preventing transmission of foot-and-mouth disease virus (O/TAW/97) by people. Veterinary microbiology 2004 v.103 no.3-4, pp. 143-149. DOI: 10.1016/j.vetmic.2004.07.020.

Arzt, J., White, W.R., Thomsen, B.V., and Brown, C.C. (2010). Agricultural diseases on the move early in the third millennium. Veterinary pathology 2010 v.47 no.1, pp. 15-27. DOI: 10.1177/0300985809354350.

Aubry, P., Thompson, J.L., Pasma, T., Furness, M.C., and Tataryn, J. (2017). Weight of the evidence linking feed to an outbreak of porcine epidemic diarrhea in Canadian swine herds. Journal of swine health and production 25, 69-72.

Ayudhya, S.N., Assavacheep, P., and Thanawongnuwech, R. (2012). One world--one health: the threat of emerging swine diseases. An Asian perspective. Transbound Emerg Dis 59 Suppl 1, 9-17. DOI: 10.1111/j.1865-1682.2011.01309.x.

Baker, K.L., Mowrer, C., Canon, A., Linhares, D.C., Rademacher, C., Karriker, L.A., et al. (2017). Systematic epidemiological investigations of cases of Senecavirus A in US swine breeding herds. Transbound Emerg Dis 64, 11-18. DOI: 10.1111/tbed.12598.

Bane, D.P., Neumann, E.J., Hall, W.F., Harlin, K.S., and Slife, R.L. (1992). Relationship between fumonisin contamination of feed and mystery swine disease. A case-control study. Mycopathologia 117, 121-124.

Beam, A., Goede, D., Fox, A., McCool, M.J., Wall, G., Haley, C., et al. (2015). A porcine epidemic diarrhea virus outbreak in one geographic region of the United States: descriptive epidemiology and investigation of the possibility of airborne virus spread. Plos one 10. DOI: 10.1371/journal.pone.0144818.

Botner, A., and Belsham, G.J. (2012). Virus survival in slurry: analysis of the stability of foot-and-mouth disease, classical swine fever, bovine viral diarrhoea and swine influenza viruses. Vet Microbiol 157, 41-49. DOI: 10.1016/j.vetmic.2011.12.010.

Bottoms, K., Dewey, C., Poljak, Z., Carter, N., and Richardson, K. (2013). Investigating disease risk associated with feed delivery. Investigating disease risk associated with feed delivery.

Brown, V.R., and Bevins, S.N. (2018). A review of African swine fever and the potential for introduction into the United States and the possibility of subsequent establishment in feral swine and native ticks. Front Vet Sci 5, 11. DOI: 10.3389/fvets.2018.00011.

Chenais, E., Sternberg-Lewerin, S., Boqvist, S., Liu, L., LeBlanc, N., Aliro, T., et al. (2017). African swine fever outbreak on a medium-sized farm in Uganda: biosecurity breaches and within-farm virus contamination. Trop Anim Health Prod 49, 337-346. DOI: 10.1007/s11250-016-1197-0.

Cochrane, R., Dritz, S., Woodworth, J., Huss, A., Stark, C., Hesse, R., et al. (2015a). Evaluating chemical mitigation of porcine epidemic diarrhea virus (PEDV) in swine feed and ingredients. Kansas Agricultural Experiment Station Research Reports 1, 5.

Cochrane, R., Schumacher, L., Dritz, S., Woodworth, J., Huss, A., Stark, C., et al. (2015b). Effect of thermal mitigation on porcine epidemic diarrhea virus (PEDV)-contaminated feed. Kansas Agricultural Experiment Station Research Reports 1, 2.

Cochrane, R.A., Dritz, S.S., Woodworth, J.C., Stark, C.R., Huss, A.R., Cano, J.P., et al. (2016a). Feed mill biosecurity plans: a systematic approach to prevent biological pathogens in swine feed. Journal of swine health and production 24, 154-164.

Cochrane, R.A., Saensukjaroenphon, M., Dritz, S.S., Woodworth, J.C., Huss, A.R., Stark, C.R., et al. (Year). "Evaluating the inclusion level of medium chain fatty acids to reduce the risk of PEDV in feed and spray-dried animal plasma", (BIOSIS Prev.).

Cochrane, R.A., Schumacher, L.L., Dritz, S.S., Woodworth, J.C., Huss, A.R., Stark, C.R., et al. (2017). Effect of pelleting on survival of porcine epidemic diarrhea virus-contaminated feed. J Anim Sci 95, 1170-1178. DOI: 10.2527/jas.2016.0961.

Dee, S., Neill, C., Clement, T., Christopher-Hennings, J., and Nelson, E. (2014). An evaluation of a liquid antimicrobial (Sal CURB(R)) for reducing the risk of porcine epidemic diarrhea virus infection of naive pigs during consumption of contaminated feed. BMC Vet Res 10, 220. DOI: 10.1186/s12917-014-0220-9.

Dee, S.A., Martinez, B.C., and Clanton, C.M. (2005). Survival and infectivity of porcine reproductive and respiratory syndrome virus in swine lagoon effluent. The Veterinary record 2005 v.156 no.2, pp. 56-57. DOI: 10.1136/vr.156.2.56.

Derbyshire, J.B., and Brown, E.G. (1978). Isolation of animal viruses from farm livestock waste, soil and water. J Hyg (Lond) 81, 295-302.

Dewey, C., Bottoms, K., Carter, N., and Richardson, K. (2014). A qualitative study to identify potential biosecurity risks associated with feed delivery. Journal of swine health and production 22, 232-243.

Disney, W.T., and Peters, M.A. (2003). Simulation modeling to derive the value-of-information for risky animal disease-import decisions. Preventive veterinary medicine 2003 v.61 no.3, pp. 171-184. DOI: 10.1016/j.prevetmed.2003.07.001.

Dougherty, R.W. (1976). Problems associated with feeding farm livestock under intensive systems. World Rev Nutr Diet 25, 249-275.

Foddai, A., Nielsen, L.H., Mogelmose, V., and Alban, L. (2015). Probability of introducing porcine epidemic diarrhea virus into Danish pig herds by imported spray-dried porcine plasma. Porcine Health Manag 1, 18. DOI: 10.1186/s40813-015-0010-1.

Gebhardt, J.T., Woodworth, J.C., Jones, C.K., Gauger, P.C., Tokach, M.D., DeRouchey, J.M., et al. (2017). "Evaluation of the effects of flushing feed manufacturing equipment with chemically-treated rice hulls on porcine epidemic diarrhea virus cross contamination during feed manufacturing", in: Annual American Association of Swine Veterinarians Meeting. (San Diego, California).

Gilchrist, M.J., Greko, C., Wallinga, D.B., Beran, G.W., Riley, D.G., and Thorne, P.S. (2007). The potential role of concentrated animal feeding operations in infectious disease epidemics and antibiotic resistance. Environ Health Perspect 115, 313-316. DOI: 10.1289/ehp.8837.

Guo, B., Lager, K.M., Henningson, J.N., Miller, L.C., Schlink, S.N., Kappes, M.A., et al. (2013). Experimental infection of United States swine with a Chinese highly pathogenic strain of porcine reproductive and respiratory syndrome virus. Virology 2013 v.435, pp. 372-384. DOI: 10.1016/j.virol.2012.09.013.

Harikumar, S., Davis, J., and Anil, K.S. (2016). Use of unconventional feed in pig production. Journal of indian veterinary association, kerala (jiva) 14, 5-11.

Hinton, M. (1993). Spoilage and pathogenic microorganisms in animal feed. International biodeterioration & biodegradation 32, 67-74.

Howey, E.B., Donnell, V., de Carvalho Ferreira, H.C., Borca, M.V., and Arzt, J. (2013). Pathogenesis of highly virulent African swine fever virus in domestic pigs exposed via intraoropharyngeal, intranasopharyngeal, and intramuscular inoculation, and by direct contact with infected pigs. ARS USDA Submissions 2013 v.178, pp. 328-339. DOI: 10.1016/j.viruses.2013.09.024.

Huss, A.R., Cochrane, R.A., Deliephan, A., Stark, C.R., and Jones, C.K. (2015). Evaluation of a biological pathogen decontamination protocol for animal feed mills. J Food Prot 78, 1682-1688. DOI: 10.4315/0362-028X.JFP-15-052.

Huss, A.R., Schumacher, L.L., Cochrane, R.A., Poulsen, E., Bai, J., Woodworth, J.C., et al. (2017). Elimination of porcine epidemic diarrhea virus in an animal feed manufacturing facility. PLoS One 12, e0169612. DOI: 10.1371/journal.pone.0169612.

Huss, A.R., Schumacher, L.L., Cochrane, R.A., Poulsen, E., Bai, J.F., Woodworth, J.C., et al. (Year). "Proof-of-concept method to sanitize a feed mill contaminated with Porcine Epidemic Diarrhea Virus", (BIOSIS Prev.), 102-103.

Kaden, V., Fischer, U., Schwanbeck, U., and Riebe, R. (1992). Is feeding of green silage in areas with hog cholera in wild boar a danger for domestic swine herds? Experimental study. Berl Munch Tierarztl Wochenschr 105, 73-77.

Kim, B., Song, J.Y., Tark, D.S., Lim, S.I., Choi, E.J., Kim, J., et al. (2008). Feed contaminated with classical swine fever vaccine virus (LOM strain) can induce antibodies to the virus in pigs. Veterinary record 162, 12-17.

Kim, Y., Yang, M., Goyal, S.M., Cheeran, M.C., and Torremorell, M. (2017). Evaluation of biosecurity measures to prevent indirect transmission of porcine epidemic diarrhea virus. BMC Vet Res 13, 89. DOI: 10.1186/s12917-017-1017-4.

Kochhar, H.S. (2014). Canada: Porcine epidemic diarrhea in Canada: an emerging disease case study. Can Vet J 55, 1048-1049.

Lowe, J., Gauger, P., Harmon, K., Zhang, J., Connor, J., Yeske, P., et al. (2014). Role of transportation in spread of porcine epidemic diarrhea virus infection, United States. Emerg Infect Dis 20, 872-874. DOI: 10.3201/eid2005.131628.

Masiuk, D.M., Sosnitsky, O.I., Nedzvetsky, V.S., Kokarev, A.V., and Koliada, S.G. (2017). Endemic course of epidemic diarrhea of pigs in the stabilized focus of infection. Regulatory Mechanisms in Biosystems 8, 410-416. DOI: 10.15421/021764.

Miao, S., and Xiao, L. (2017). Study on basic level animal epidemic prevention system - taking swine epidemic diseases as an example. Animal husbandry and feed science (Inner Mongolia) 38, 92-96.

Moore, C. (1992). Biosecurity and minimal disease herds. Vet Clin North Am Food Anim Pract 8, 461-474.

Moreno-López, J. (2002). Contaminants in feed for food-producing animals. Polish journal of veterinary sciences 5, 123-125.

Morgavi, D.P., and Riley, R.T. (2007). An historical overview of field disease outbreaks known or suspected to be caused by consumption of feeds contaminated with Fusarium toxins. Animal feed science and technology 2007 v.137 no.3-4, pp. 201-212. DOI: 10.1016/j.anifeedsci.2007.06.002.

Muroga, N., Kobayashi, S., Nishida, T., Hayama, Y., Kawano, T., Yamamoto, T., et al. (2013). Risk factors for the transmission of foot-and-mouth disease during the 2010 outbreak in Japan: a case–control study. BMC veterinary research 2013 v.9 no.1, pp. 714-714. DOI: 10.1186/1746-6148-9-150.

Nugent, R. (Year). "State of the knowledge: the relationships between PEDV/PDCoV transmission and feed", in: AASV Annual Meeting. American Association of Swine Veterinarians).

Pasick, J., Berhane, Y., Ojkic, D., Maxie, G., Embury-Hyatt, C., Swekla, K., et al. (2014). Investigation into the role of potentially contaminated feed as a source of the first-detected

outbreaks of porcine epidemic diarrhea in Canada. Transbound Emerg Dis 61, 397-410. DOI: 10.1111/tbed.12269.

Pasma, T., Furness, M.C., Alves, D., and Aubry, P. (2016). Outbreak investigation of porcine epidemic diarrhea in swine in Ontario. Can Vet J 57, 84-89.

Perri, A.M., Poljak, Z., Dewey, C., Harding, J.C.S., and O'Sullivan, T.L. (2018). An epidemiological investigation of the early phase of the porcine epidemic diarrhea (PED) outbreak in Canadian swine herds in 2014: A case-control study. Prev Vet Med 150, 101-109. DOI: 10.1016/j.prevetmed.2017.12.009.

Pitkin, A., Deen, J., and Dee, S. (2009). Further assessment of fomites and personnel as vehicles for the mechanical transport and transmission of porcine reproductive and respiratory syndrome virus. Can J Vet Res 73, 298-302.

Polo, J., Quigley, J.D., Russell, L.E., Campbell, J.M., Pujols, J., and Lukert, P.D. (2005). Efficacy of spray-drying to reduce infectivity of pseudorabies and porcine reproductive and respiratory syndrome (PRRS) viruses and seroconversion in pigs fed diets containing spray-dried animal plasma. J Anim Sci 83, 1933-1938. DOI: 10.2527/2005.8381933x.

Pujols, J., Rodriguez, C., Navarro, N., Pina-Pedrero, S., Campbell, J.M., Crenshaw, J., et al. (2014). No transmission of hepatitis E virus in pigs fed diets containing commercial spray-dried porcine plasma: a retrospective study of samples from several swine trials. Virol J 11, 232. DOI: 10.1186/s12985-014-0232-x.

Pyburn, D. (1998). Feed safety in the swine industry. Proceedings one hundred and second annual meeting of the united states animal health association, minneapolis, minnesota, usa, 3-9 october, 1998, 208-209.

Quist-Rybachuk, G.V., Nauwynck, H.J., and Kalmar, I.D. (2015). Sensitivity of porcine epidemic diarrhea virus (PEDV) to pH and heat treatment in the presence or absence of porcine plasma. Vet Microbiol 181, 283-288. DOI: 10.1016/j.vetmic.2015.10.010.

Ribbens, S., Dewulf, J., Koenen, F., Laevens, H., and de Kruif, A. (2004). Transmission of classical swine fever. A review. Vet Q 26, 146-155. DOI: 10.1080/01652176.2004.9695177.

Rose, N., and Grasland, B. (2014). Porcine epidemic diarrhea virus. Le nouveau praticien vétérinaire élevages et santé, 48-55.

Rose, N., Opriessnig, T., Grasland, B., and Jestin, A. (2012). Epidemiology and transmission of porcine circovirus type 2 (PCV2). Virus research 2012 v.164 no.1-2, pp. 78-89. DOI: 10.1016/j.virusres.2011.12.002.

Rossi, S., Fromont, E., Pontier, D., Crucière, C., Hars, J., Barrat, J., et al. (2003). Interactions between classical swine fever virus and wild boar (Sus scrofa) in France; Ten years of survey: 1992-2002. Interactions between classical swine fever virus and wild boar (sus scrofa) in france; ten years of survey: 1992-2002.

Schumacher, L.L., Cochrane, R.A., Evans, C.E., Kalivoda, J.R., Woodworth, J.C., Huss, A.R., et al. (Year). "Evaluating the effect of manufacturing porcine epidemic diarrhea virus (PEDV)-contaminated feed on subsequent feed mill environmental surface contamination", (BIOSIS Prev.).

Schumacher, L.L., Cochrane, R.A., Woodworth, J.C., Huss, A.R., Stark, C.R., Jones, C.K., et al. (Year). "Utilizing feed sequencing to decrease the risk of porcine epidemic diarrhea virus (PEDV) cross-contamination during feed manufacturing", (BIOSIS Prev.).

Schumacher, L.L., Huss, A.R., Cochrane, R.A., Stark, C.R., Woodworth, J.C., Bai, J., et al. (2017). Characterizing the rapid spread of porcine epidemic diarrhea virus (PEDV) through an animal food manufacturing facility. PLoS One 12, e0187309. DOI: 10.1371/journal.pone.0187309.

Schumacher, L.L., Woodworth, J.C., Stark, C.R., and Jones, C.K. (2015). Determining the minimum infectious dose of porcine epidemic diarrhea virus (PEDV) in a feed matrix. Journal Volume 1, Issue 7 Swine Day.

Sindryakova, I.P., Morgunov, Y.P., Chichikin, A.Y., Gazaev, I.K., Kudryashov, D.A., and Tsybanov, S.Z. (2016). The influence of temperature on the Russian isolate of African swine fever virus in pork products and feed with extrapolation to natural conditions. Sel'skokhozyaistvennaya Biologiya 51, 467-474. DOI: 10.15389/agrobiology.2016.4.467eng.

Snider, T., and Smith, J.H. (2015). PEDV and the feed supply chain - risk and biosecurity. Proceedings of the 15th London Swine Conference: Production Technologies to Meet Market Demands, 1-2 April 2015, London, Ontario, Canada.

Stadler, J., Zoels, S., Fux, R., Hanke, D., Pohlmann, A., Blome, S., et al. (2015). Emergence of porcine epidemic diarrhea virus in southern Germany. BMC veterinary research 11, 142-142. DOI: 10.1186/s12917-015-0454-1.

Steinrigl, A., Revilla Fernández, S., Stoiber, F., Pikalo, J., Sattler, T., and Schmoll, F. (2015). First detection, clinical presentation and phylogenetic characterization of porcine epidemic diarrhea virus in Austria. BMC veterinary research 11, 310-310. DOI: 10.1186/s12917-015-0624-1.

Trudeau, M.P., Verma, H., Sampedro, F., Urriola, P.E., Shurson, G.C., McKelvey, J., et al. (2016). Comparison of thermal and non-thermal processing of swine feed and the use of selected feed additives on inactivation of porcine epidemic diarrhea virus (PEDV). Plos one 11.

Truszczynski, M., and Pejsak, Z. (2014). Coronaviruses and diseases which they cause in swine. Medycyna weterynaryjna-veterinary medicine-science and practice 70, 131-135.

Xiao, P., Li, R., She, R., Yin, J., Li, W., Mao, J., et al. (2012). Prevalence of hepatitis E virus in swine fed on kitchen residue. PLoS One 7, e33480. DOI: 10.1371/journal.pone.0033480.
