# Supplementary material for: The Role of Non-animal Origin Feed Ingredients in Transmission of Viral Pathogens of Swine: A Review of Scientific Literature
Source: Front Vet Sci. 2019 Aug 22;6:273. doi: 10.3389/fvets.2019.00273 (PMC6714588; doi:10.3389/fvets.2019.00273)
Supplement: Supplementary file 3 [file Data_Sheet_2.PDF]

Data Sheet S2: Studies included (n = 26) in the literature review

Bowman, A.S., Krogwold, R.A., Price, T., Davis, M., and Moeller, S.J. (2015). Investigating the introduction of porcine epidemic diarrhea virus into an Ohio swine operation. BMC veterinary research 2015 v.11 no.1, pp. 38-38. DOI: 10.1186/s12917-015-0348-2.

Brookes, V.J., Hernandez-Jover, M., Holyoake, P., and Ward, M.P. (2015). Industry opinion on the likely routes of introduction of highly pathogenic porcine reproductive and respiratory syndrome into Australia from south-east Asia. Aust Vet J 93, 13-19. DOI: 10.1111/avj.12284.

Davies, P.R. (2015). The dilemma of rare events: Porcine epidemic diarrhea virus in North America. Prev Vet Med 122, 235-241. DOI: 10.1016/j.prevetmed.2015.08.006.

Dee, S., Clement, T., Schelkopf, A., Nerem, J., Knudsen, D., Christopher-Hennings, J., et al. (2014). An evaluation of contaminated complete feed as a vehicle for porcine epidemic diarrhea virus infection of naïve pigs following consumption via natural feeding behavior: proof of concept. Bmc veterinary research 10.

Dee, S., Neill, C., Clement, T., Singrey, A., Christopher-Hennings, J., and Nelson, E. (2015). An evaluation of porcine epidemic diarrhea virus survival in individual feed ingredients in the presence or absence of a liquid antimicrobial. Porcine health management 1, 9. DOI: 10.1186/s40813-015-0003-0.

Dee, S., Neill, C., Singrey, A., Clement, T., Cochrane, R., Jones, C., et al. (2016). Modeling the transboundary risk of feed ingredients contaminated with porcine epidemic diarrhea virus. Bmc veterinary research 12, 51. DOI: 10.1186/s12917-016-0674-z.

Dee, S.A., Bauermann, F.V., Niederwerder, M.C., Singrey, A., Clement, T., de Lima, M., et al. (2018). Survival of viral pathogens in animal feed ingredients under transboundary shipping models. PLoS One 13, e0194509. DOI: 10.1371/journal.pone.0194509.

EFSA (European Food Safety Authority) (2016). Scientific report on the collection and review of updated epidemiological data on porcine epidemic diarrhoea. 14(2), 4375. DOI: 10.2903/j.efsa.2016.4375.

EFSA AHAW Panel (EFSA Panel on Animal Health and Welfare) (2014). Scientific Opinion on porcine epidemic diarrhoea and emerging porcine deltacoronavirus. EFSA Journal 12(10), 3877. DOI: 10.2903/j.efsa.2014.3877.

Fasina, F.O., Agbaje, M., Ajani, F.L., Talabi, O.A., Lazarus, D.D., Gallardo, C., et al. (2012). Risk factors for farm-level African swine fever infection in major pig-producing areas in Nigeria, 1997-2011. Prev Vet Med 107, 65-75. DOI: 10.1016/j.prevetmed.2012.05.011.

Goyal, S.M. (2014). Environmental stability of PEDV (porcine epidemic diarrhea virus). Research Report Swine Health.

Greiner, L.L. (2016). Evaluation of the likelihood of detection of porcine epidemic diarrhea virus or porcine delta coronavirus ribonucleic acid in areas within feed mills. Journal of swine health and production 24, 198-204.

Guinat, C., Gogin, A., Blome, S., Keil, G., Pollin, R., Pfeiffer, D.U., et al. (2016). Transmission routes of African swine fever virus to domestic pigs: current knowledge and future research directions. Veterinary record 178, 262-267. DOI: 10.1136/vr.103593.

Le, H., Poljak, Z., Deardon, R., and Dewey, C.E. (2012). Clustering of and risk factors for the porcine high fever disease in a region of Vietnam. Transbound Emerg Dis 59, 49-61. DOI: 10.1111/j.1865-1682.2011.01239.x.

Lowe, J.F. (2014). Porcine epidemic diarrhoea virus in the USA: lessons learned from the 2013 outbreak. Cab reviews 9, 1-4. DOI: 10.1079/PAVSNNR20149042.

Martinez-Gamba, R., P., P.-R., P., C.F., M., H., E., G., and C., M. (2001). Persistence of Escherichia coli, Salmonella choleraesuis, Aujeszky's Disease virus and Blue Eye Disease virus in ensilages based on the solid fraction of pig faeces. Journal of applied microbiology 91, 750-758.

McCluskey, B.J., Haley, C., Rovira, A., Main, R., Zhang, Y., and Barder, S. (2016). Retrospective testing and case series study of porcine delta coronavirus in U.S. swine herds. Preventive veterinary medicine 2016 v.123, pp. 185-191. DOI: 10.1016/j.prevetmed.2015.10.018.

Niederwerder, M.C., and Hesse, R.A. (2018). Swine enteric coronavirus disease: A review of 4 years with porcine epidemic diarrhoea virus and porcine deltacoronavirus in the United States and Canada. Transboundary and emerging diseases.

Pillatzki, A.E., Gauger, P.C., Madson, D.M., Burrough, E.R., Zhang, J., Chen, Q., et al. (2015). Experimental inoculation of neonatal piglets with feed naturally contaminated with porcine epidemic diarrhea virus. Journal of swine health and production 23, 317-320.

Pirtle, E.C., and Beran, G.W. (1996). Stability of porcine reproductive and respiratory syndrome virus in the presence of fomites commonly found on farms. J Am Vet Med Assoc 208, 390-392.

Sasaki, Y., Alvarez, J., Sekiguchi, S., Sueyoshi, M., Otake, S., and Perez, A. (2016). Epidemiological factors associated to spread of porcine epidemic diarrhea in Japan. Preventive veterinary medicine 123, 161-167. DOI: 10.1016/j.prevetmed.2015.11.002.

Schoenbaum, M.A., Freund, J.D., and Beran, G.W. (1991). Survival of pseudorabies virus in the presence of selected diluents and fomites. Journal of the american veterinary medical association 198, 1393-1397.

Schumacher, L.L., Woodworth, J.C., Jones, C.K., Chen, Q., Zhang, J., Gauger, P.C., et al. (2016). Evaluation of the minimum infectious dose of porcine epidemic diarrhea virus in virus-inoculated feed. Am J Vet Res 77, 1108-1113. DOI: 10.2460/ajvr.77.10.1108.

Scott, A., McCluskey, B., Brown-Reid, M., Grear, D., Pitcher, P., Ramos, G., et al. (2016). Porcine epidemic diarrhea virus introduction into the United States: root cause investigation. Preventive veterinary medicine 123, 192-201. DOI: 10.1016/j.prevetmed.2015.11.013.

Trudeau, M.P., Harsha, V., Urriola, P.E., Sampedro, F., Shurson, G.C., and Goyal, S.M. (2017b). Survival of Porcine Epidemic Diarrhea Virus (PEDV) in thermally treated feed ingredients and on surfaces. Porcine health management 3.

Trudeau, M.P., Verma, H., Sampedro, F., Urriola, P.E., Shurson, G.C., and Goyal, S.M. (2017a). Environmental persistence of porcine coronaviruses in feed and feed ingredients. Plos one 12. DOI: 10.1371/journal.pone.0178094.
