# Supplementary material for: The Role of Non-animal Origin Feed Ingredients in Transmission of Viral Pathogens of Swine: A Review of Scientific Literature
Source: Front Vet Sci. 2019 Aug 22;6:273. doi: 10.3389/fvets.2019.00273 (PMC6714588; doi:10.3389/fvets.2019.00273)
Supplement: Supplementary file 4 [file Data_Sheet_3.pdf]

Data Extraction Form for Non-animal Origin Feed Ingredient (NOFI) Literature Review

General Information

1. Reviewer(s):
2. Date of data extraction:
3. Author(s):
4. Article Title:
5. Type of publication:

☒ No Selection ☐ Journal article ☐ Government publication
☐ Conference abstract ☐ Other (specify)

6. Publication name/year:
7. Country of origin:
8. Source of funding/author affiliation:

Study Characteristics

1. Study objectives:
2. Study design:
3. Study inclusion and exclusion criteria:
4. Unit of allocation:
5. Study description (brief)

Outcome data/results

1. Unit of assessment/analysis

2. Statistical techniques used:

3. Results of study:

Quality Assessment

1. Appropriateness of study design to the research objective:

2. Sources of potential bias:

3. Statistical/analysis issues:

4. Quality of reporting:

5. Generalizability:

6. Overall quality:

■ No selection ☐ High ☐ Medium ☐ Low

7. Comments:

Figure S1. Data extraction for template
